# Whose shoulders is health research standing on? Determining the key actors and contents of the prevailing biomedical research agenda

**DOI:** 10.1371/journal.pone.0249661

**Published:** 2021-04-07

**Authors:** Federico E. Testoni, Mercedes García Carrillo, Marc-André Gagnon, Cecilia Rikap, Matías Blaustein

**Affiliations:** 1 Facultad de Filosofía y Letras (FFyL), Instituto de Lingüística, Universidad de Buenos Aires (UBA), Buenos Aires, Argentina; 2 Facultad de Ciencias Exactas y Naturales (FCEyN), Departamento de Fisiología, Biología Molecular y Celular (DFBMC), Instituto de Biociencias, Biotecnología y Biología Traslacional (iB3), Universidad de Buenos Aires (UBA), Buenos Aires, Argentina; 3 School of Public Policy and Administration, Carleton University, Ottawa, Canada; 4 Consejo Nacional de Investigaciones Científicas y Técnicas (CONICET), Argentina; 5 CEPED, IRD/Université Paris Descartes, Université Sorbonne Paris Cité, Paris, France; University of Toronto, CANADA

## Abstract

**Background:**

Conflicts of interest in biomedical research can influence research results and drive research agendas away from public health priorities. Previous agenda-setting studies share two shortfalls: they only account for direct connections between academic institutions and firms, as well as potential bias based on researchers’ personal beliefs. This paper’s goal is to determine the key actors and contents of the prevailing health and biomedical sciences (HBMS) research agenda, overcoming these shortfalls.

**Methods:**

We performed a bibliometric and lexical analysis of 95,415 scientific articles published between 1999 and 2018 in the highest impact factor journals within HBMS, using the Web of Science database and the CorText platform. HBMS’s prevailing knowledge network of institutions was proxied with network maps where nodes represent affiliations and edges the most frequent co-authorships. The content of the prevailing HBMS research agenda was depicted through network maps of prevalent multi-terms found in titles, keywords, and abstracts.

**Results:**

The HBMS research agendas of large private firms and leading academic institutions are intertwined. The prevailing HBMS agenda is mostly based on molecular biology (40% of the most frequent multi-terms), with an inclination towards cancer and cardiovascular research (15 and 8% of the most frequent multi-terms, respectively). Studies on pathogens and biological vectors related to recent epidemics are marginal (1% of the most frequent multi-terms). Content of the prevailing HBMS research agenda prioritizes research on pharmacological intervention over research on socio-environmental factors influencing disease onset or progression and overlooks, among others, the study of infectious diseases.

**Conclusions:**

Pharmaceutical corporations contribute to set HBMS’s prevailing research agenda, which is mainly focused on a few diseases and research topics. A more balanced research agenda, together with epistemological approaches that consider socio-environmental factors associated with disease spreading, could contribute to being better prepared to prevent and treat more diverse pathologies and to improve overall health outcomes.

## Introduction

The influence of industry over scientific research raises many debates, particularly when it comes to setting research agendas. Some empirical analyses found that collaborations between industry and university generate a skewing problem (or dilemma), by creating a bias in academic agendas towards private needs [1,2]. Other authors argued, on the contrary, that agendas have not been significantly skewed due to the commercialization of research and its outcomes [3–5].

Regardless of their different results, all previous investigations share two problematic features. First, their chosen methodology is either based on surveys or interviews with different stakeholders (such as researchers and university authorities) or consists of case studies. Searching for a skewing problem by analysing researchers’ impressions presents a shortfall since, as was observed by Kleinman and Vallas [6], surveyed or interviewed researchers may ignore external influences that affect their research agenda. Second, they look at the explicit influence of research commercialization on the determination of academic research agendas by analysing direct links with private corporations (through private sponsorship of academic research, agreements, exchanges in academic events, or other informal knowledge transfers, among others). This approach may cause the interests of corporations to influence the non-directly related research agendas of academic institutions. For instance, when a leading academic institution establishes direct links with both private stakeholders and other academic institutions, the institution may end up unwillingly transferring the research priorities of the former to the latter.

In the specific case of medical research, there is a more unanimous agreement on the impact of industry sponsorship and conflicts of interest in medical research and clinical practice, which ultimately influence research results [7–9]. A different question, however, is if, and the extent to which private firms can influence research agendas and set priorities in the prevailing network of medical knowledge production. Fabbri et al. offered an in-depth review of medical literature, showing that corporate interests can drive research agendas away from issues that are more relevant to public health [10]. Previous studies also show that the pharmaceutical industry’s expenses on marketing and promotional activities predominate over research and development (R&D) expenses [11]. Unsurprisingly, corporations tend to fund research in areas that guarantee a large market share. This is able to explain examples such as pharmaceutical companies being more likely to sponsor studies on diseases that affect high-income countries [12,13]. Whether this corporate influence over academic research agendas represents a phenomenon observed at the margin of health and biomedical sciences (HBMS) investigations or sets the prevailing HBMS research agenda remains an open question. We addressed this question with an interdisciplinary approach, building on the theoretical concept of agenda from the agenda-setting theory [14]. This theory focuses on the salience of objects (things that an individual has an attitude or an opinion about) within media discourse and the consequences that this has on general public agendas. The proposed study focuses on the content of these agendas, rather than the efforts to dictate them (e.g., in our case, public policies regarding HBMS) and the projection of that kind of study to different agendas in contemporary society [14]. This way, we are able to reconstruct a specific agenda from the different levels of presence that different topics have in the outcome of a practice, in this case, the presence of different terms associated with problems and methodologies in HBMS scientific research.

The field of HBMS constitutes a model case of blurred boundaries between academic and commercial research [15], and thus represents an ideal system to investigate the centrality of corporate interest on the prevailing research agenda. This analysis is especially relevant in the context of the exhaustion of the blockbuster business model and the recent transformation of innovation strategies focusing on specialized medicine, in which pharmaceutical companies intensified the outsourcing of certain stages of their R&D process [16–18]. As a result, large pharmaceuticals organize, steer, and ultimately control global innovation networks, where leading universities and other academic research organizations are active participants [19,20].

To proxy the HBMS research agenda, and search for a potential influence of private corporations in setting that agenda, we analysed scientific publications, the most common academic research outcome. Within the overall set of publications in a field, those in high-impact factor journals exert the greatest influence in terms of that field’s research priorities. Therefore, we defined the “prevailing HBMS research agenda” as the agenda characterized by the prevalent multi-terms found in the 30 journals with the highest impact factors within the HBMS field. This approach allowed us to identify key actors and contents of that agenda, excluding previous shortfalls, as well as to examine three issues: (1) Whether private corporations participate in the HBMS’s prevailing network of research organizations; (2) Whether the prevailing HBMS research agenda involves the study of a diversity of diseases, or if there is an inclination toward specific ones; and (3) Whether the prevailing HBMS research agenda involves a plurality of research topics and methodologies.

## Materials and methods

### Search strategy and selection criteria

To conduct our investigation, we retrieved a corpus of HBMS scientific publications from Web of Science (WoS) that proxies the prevailing HBMS research agenda. WoS classifies journals in terms of scientific categories, so we manually selected all the specific categories that corresponded to HBMS (listed in S1 Table legend). Next, we retrieved the full list of journals that corresponded to any of those specific categories and selected the 30 journals with the highest impact factors. For the selected journals, we retrieved all available publications (including original research articles, reviews, perspectives, editorials, etc.) between 1999 and 2018: 96,045 papers, out of which we were able to analyse 95,415 (those articles from which we could not gather all the required information or displayed errors during processing—0.7% from the total—were filtered). This provided a corpus to investigate the prevailing HBMS research agenda.

To assess the temporal evolution of this research agenda, we split the corpus into two regular sub-periods: 1999–2008 and 2009–2018. The sub-periods were relatively homogeneous in terms of the number of publications (47,958 and 47,457 papers, respectively), indicating that the publishing frequency of top journals remained stable.

### Data analysis

The data was processed using the CorText platform [21], which allowed us to build co-occurrence maps. These maps were constructed by using specific algorithms that associate entities (names of research institutions and most frequent terms) according to their frequency of co-occurrence within a chosen corpus of texts [22]. In our analysis, the corpus consisted of a set of scientific publications. The procedure used to draw these maps, including the filtering of the corpus, followed the methodology presented in Tancoigne et al. [21].

Given our research question, we followed a two-step process: 1) we reconstructed the HBMS network of prevailing publishing institutions, defined as those that co-occurred with the highest frequency as authors’ affiliations in our corpus, and 2) we analysed the prevailing content of the research included in our corpus, proxying the prevailing research agenda. For each step, we introduced a dynamic component by considering the two mentioned periods. A step-by-step methodology is provided in the following section.

#### 1) Reconstruction of the prevailing HBMS knowledge network

Unlike other bibliometric databases, the WoS provides a separate field with authors’ affiliations called “research institution”. For each of the two selected periods, we used this field to map the most frequently connected affiliations.

To build these network maps, we based our methodology on previous work. These previous studies showed that social network analyses using co-patenting and co-publication data allow for mapping relations between actors within a knowledge or innovation system [23,24]. This has become a standard way of measuring science-industry collaborations [25]. Cooke [26], for example, analysed top impact factor journals from HBMS to proxy its prevailing knowledge network. However, this study focused on the geographical distribution of this network, overlooking the specific actors involved and their research agenda(s).

Although the WoS presents an already cleaned database, affiliations frequently appeared spelled differently. To build a harmonized list, thus assuring that each institution appeared under only a single name, we followed the methodology presented in Tancoigne et al. [21]. First, we listed all institutions in alphabetical order and created a new field with a unified affiliation for each institution. We defined special criteria to determine each type of institution. In our retrieved corpus, each university was renamed as “univ” followed by the rest of its name in English. University hospitals and schools were renamed after the name of their corresponding university. For all the hospitals with names that did not indicate a university affiliation, we searched on Wikipedia and the hospital’s website to find possible affiliations. To unify the names of private firms when they appeared as affiliations, we worked with companies’ (in particular large pharmaceuticals) corporate trees, as provided by Derwent Innovation. Furthermore, in the United States (US), United Kingdom (UK) and France, some institutions belong to a “university system” (called COMUE in France). As a result, many researchers only include their university system affiliation and others add the name of their particular institution within that grouping. To have a unified criterion for every organization and considering the corpus limitations (it was not always possible to disentangle the institutions), we followed Rikap [20] and merged all the affiliations at the level of the corresponding parent organization.

The simplest network of research collaboration between institutions appearing in the field “research institutions” in our corpus would have been one that _consider_s all the institutions that have collaborated at one time or another as connected by a link. But the resulting network would have been too dense and not very informative. Since we wanted to focus on the most influential institutions (defined as those with the highest publishing frequency at each time period), we prioritized the top 200 nodes or vertices. To weight the network’s links, we used chi-square proximity measure to determine nodes and edges to be considered in each network map. Hence, the applied chi-square metric to construct the co-occurrence matrices of each of our network analyses was defined as:

$${S_{x^{2}}(i,\;j)}_{\;}=\frac{c_{ij}{-\;e}_{ij}}{\sqrt{e_{ij}}}$$ (1)

where c_ij_ is the number of joint occurrences of *i* and *j* in the same document (in our case in the same scientific publication) and e_ij_ is the expected number of co-occurrences. e_ij_ is defined as the total number of co-occurrences of i (s_i_) multiplied by the total number of co-occurrences of j (s_j_) divided by the global number of co-occurrences (N):

$$e_{ij}=\frac{s_{i}\;s_{j}}{N}\;\text{where}\;\text{N}=\sum_{i}s_{i}$$ (2)

Chi-square is a direct local measure, meaning that it considers actual occurrences between entities. Indirect measures, such as the distributional one, build network maps based on the similarity of two nodes in comparison with their entire co-occurrence profile with the other identified entities [21]. Hence, they should not be applied in our case because our research question requires looking for actual links. Chi-square normalization tends to create links towards higher degree nodes. Therefore, this metric prioritizes the most frequent co-occurrences within the network and those are the direct ties (edges). Applied to our corpus, using the chi-square measure creates direct connections only for the most frequently co-authoring institutions from total co-authorships.

Next, we used the Louvain community detection algorithm as our cluster detection method [27]. A cluster means that in relation to the rest of the edges, those within the cluster represent the most frequent co-occurrences of the nodes that belong to that cluster.

**Fig 1:**
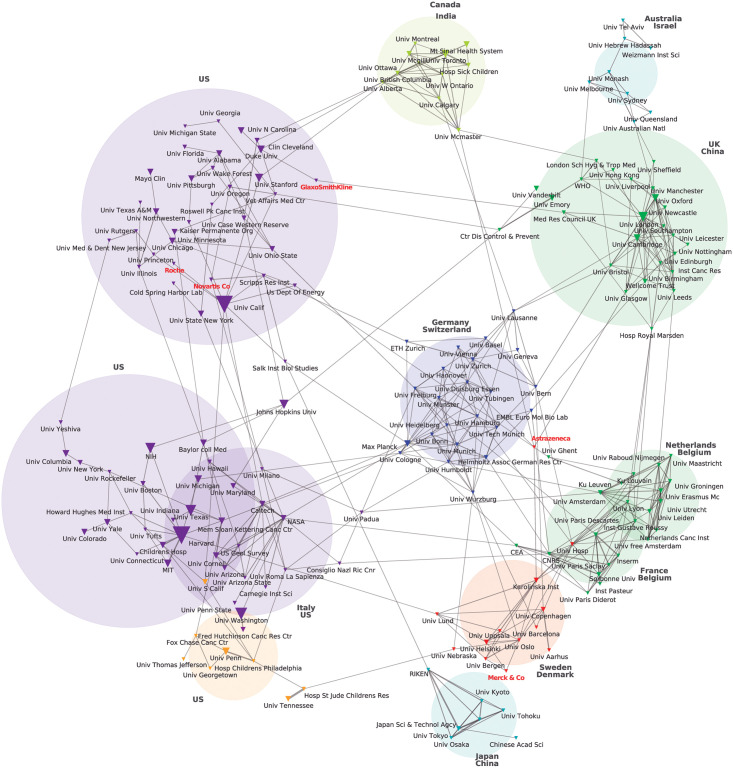
Top 200 health and biomedical sciences (HBMS) research affiliations (1999 to 2008) plotted according to co-authorship using a chi-squared distribution. Source: Authors’ analysis based on Web of Science (WoS) data extraction plotted via CorTexT.

**Fig 2:**
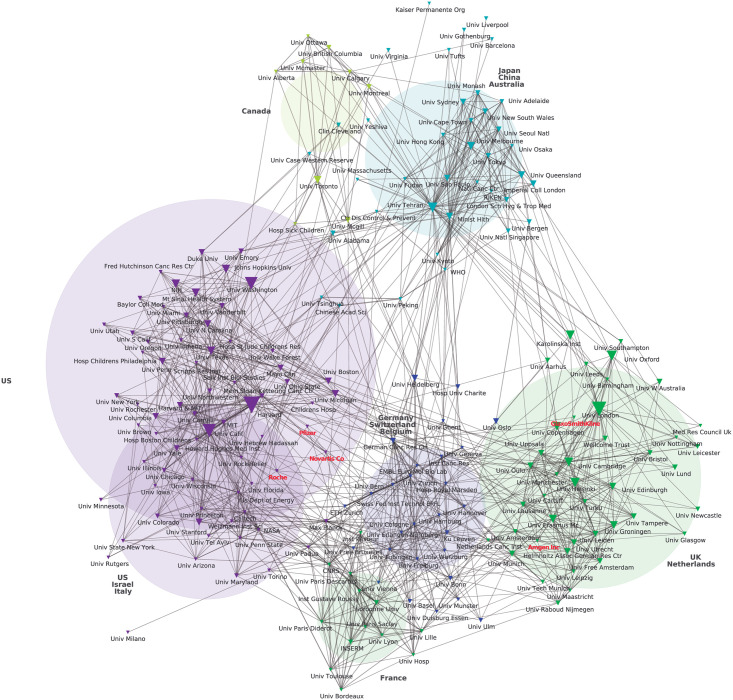
Top 200 HBMS research affiliations (2009 to 2018) plotted according to co-authorship using a chi-squared distribution. Source: Authors’ analysis based on WoS data extraction plotted via CorTexT.

In the resulting maps (Figs 1 and 2), the nodes represent institutions (universities, research bodies, enterprises, etc.). The size of the nodes represents the frequency of each institution’s appearance in the dataset (thus each institution’s publishing frequency for selected journals).

As previously explained, we used chi-square, which is a normalized proximity measure that creates links (edges) towards higher degree nodes. Edges connecting nodes or vertices represent the most frequent (but not all) co-authorships. When two nodes were indirectly linked by a third node, in the network, this third institution occupied a bridging position.

A standard measure for considering the intermediating role of each node in a network is that of betweenness centrality, defined as the sum of the ratio of the shortest paths between any two nodes in the network that pass through that node. We calculated this measure using Gephi and considered it to evaluate the position of the large pharmaceuticals that appeared in Figs 1 and 2. Besides this measure, since we grouped nodes in clusters, we were also interested in the number of clusters that a single node connects as an indicator of a research institutions’ influence in the overall network. A node could have a high betweenness centrality but only because it occupies a central position inside its cluster. In this case and considering our research problem, that research institution could be interpreted as having a high influence in setting the research agenda of its cluster, but the measure would not say much of its chance of transferring its R&D priorities to the general network. This is why we complemented this measure with an analysis of the number of edges, in particular edges with nodes from different clusters, considering the latter as bridges between clusters.

Nodes occupying bridging positions connecting different clusters are of particular relevance for holding the clusters together and “in the dynamics of spreading processes across the network” [28]. Bridges represent channels through which research priorities can be established and disseminated in both directions. If a public institution or a private firm occupies a bridging position, it will have a better chance of transferring its R&D priorities to the general network. In other words, the chances of exerting indirect influence will be greater in a bridging position than in a position with very few edges within the same group.

The resulting network maps -one for each period- can be conceived as a proxy of HBMS’s prevailing knowledge network of organizations.

#### 2) Reconstruction of HBMS top journals’ research agenda

The definition of “agenda” that we have used conceives agendas as flowing from the set of prevailing topics shared by a community. In our case, the agenda is defined as the prevailing topics of the HBMS research community. Furthermore, by focusing on top journals by impact factor to proxy this agenda, we are assuming that topics most frequently studied in these journals’ publications influence the overall HBMS research community. In other words, we assume a normative influence of high impact factor journals over the whole research community within a research field [29,30]. Since these journals have the highest impact factors, it means that on average, each journal’s publications are the most cited ones. This is an indication of their greater relative influence within the HBMS field.

To build the prevailing HBMS research agenda, we performed a lexical analysis of the titles, keywords, and abstracts of the full HBMS high-impact factor journals’ corpus. We extracted the top 500 multi-terms of up to 5 words as a proxy of privileged topics. Monograms were excluded, and each list was refined following an in-depth cleaning process. This filtering was performed to avoid words not related to the HBMS field and whose frequency responds to either their grammatical function (such as “and” and “or”) or the level of grammaticalization within the scientific genre (“methods and results”, “case study”, “present study”, “positive effect”, etc.). The resulting list of 320 multi-terms was classified into general categories according to research topics (such as “Cancer/Tumor” or “Cardiovascular”). During the process of selecting the key multi-terms, we found that many were linked to methods and procedures. We decided to include them, as they provide valuable data on the nature of the research under analysis. For instance, a paper may contain terms such as “polymerase chain reaction” or “electron microscopy” in its title and/or abstract, which may indicate that the topic is being studied from a molecular and cellular biology perspective and/or using tools associated with this specialty.

A network map was built for each period, plotting its corresponding most frequently connected multi-terms, following the same procedure used for research institutions’ network maps. In this case, nodes represented multi-terms. We prioritized the top 100 for each period (similar overall results were obtained selecting the top 150 multi-terms for each period). These maps also included the general categories’ classification that we made as a third depicted variable. We plotted the top three general categories associated with each cluster. We inferred that the most frequently connected multi-terms corresponded to those research topics and methodologies that define the prevailing HBMS research agenda. As explained by Barbier et al. [22], “co-word analysis maps various types of associations between terms that ontologically represent the textual strategizing of authors is a method that extracts data from texts without presumptions about their content”. For the same reasons explained above, we also used chi-square to determine the edges of the multi-terms’ network maps and the Louvain algorithm to detect clusters.

## Results

### HBMS prevailing network of knowledge production

To proxy the HBMS prevailing research agenda and to identify the possible presence of corporate influence, we applied bibliometric and lexical analysis techniques to a corpus of 95,415 scientific publications from the 30 highest impact factor journals within HBMS (S1 Table) between 1999 and 2018. Briefly, we assessed the number of publications authored by each organization and mapped the most frequently connected co-authors’ affiliations in this corpus of scientific publications.

Our results show that HBMS’s prevailing network of research organizations is led both by prominent academic research institutions and large pharmaceutical corporations (Figs 1 and 2; S2 Table). This means that these corporations are among the top 200 organizations in terms of co-occurrence frequency in our corpus of the 30 journals with the highest impact factors within the HBMS field. As we show in Fig 1, where we consider co-authorship frequency for the first period (1999–2008), there are large pharmaceutical corporations among the HBMS’s prevailing network of research organizations, namely: Roche, Merck, Novartis, AstraZeneca, and GlaxoSmithKline (Fig 1).

In the second period (2009–2018), Merck and AstraZeneca disappear from the network, and Pfizer and Amgen show up (Fig 2). In comparison with the first period, large pharmaceutical companies increased connections with other institutions of the network -from 15 total direct links (3 on average) to 32 (6 on average)- pointing to a growing capacity to influence HBMS’s prevailing research agenda. Particularly, Roche’s bridging position in the second period stands out. First, its betweenness centrality went from 0 in the first period to 0.003 in the second period. This change represented a higher rank according to this measure, and thus a relatively greater intermediator role. Second, from having only one direct link within the same cluster (Fig 1), Roche connects with 11 institutions from 4 different clusters in the second period (Fig 2). This result is of particular relevance if we consider, for instance, that in the same period the University of London, that has the 3^rd^ highest betweenness centrality (0.062), was directly connected to nodes corresponding to 3 different clusters. Moreover, Novartis was ranked 112 in betweenness centrality in the first period and 57 in the second (from 0.0058 to 0.0089).

The ranking of organizations in terms of overall publishing frequency in distinct documents provides similar results for the whole period (1999–2018). Roche, GlaxoSmithKline, Pfizer, and Merck together with Amgen Inc. occupy positions between the 64^th^ and the 200^th^ (S2 Table).

In terms of the geopolitical distribution of HBMS’s prevailing knowledge network, leading countries and regions stand out. In the first period, different clusters can be associated with a variety of countries. However, 4 clusters (a 25% overall) are linked to the US (this means that most of the nodes correspond to US-based organizations). Particularly, Roche and Novartis always appear in US-dominated clusters even if they are Swiss in origin. Within Europe, the UK, Germany, France, Italy, Netherlands, Switzerland, Belgium, Sweden, and Denmark predominate (Fig 1).

In the second period, the US (36%), the UK (11%), Germany (10%), and France (6%) concentrate over 60% of total institutions. Other 25 countries are represented in the corresponding map, but with marginal participation (Fig 2). Seven of the top ten publishing organizations throughout our whole study period are based in the US according to their publishing frequency (S2 Table). The world’s top two organizations are Harvard and the University of California (12,067 and 11,090 different scientific publications between 1999 and 2018, respectively). They each occupy a central position in a different cluster and occupy bridging network positions in both periods (S2 Table). The top ten is completed with two universities from the UK (the University of London in the 3^rd^ position, and Oxford University in the 9^th^ position) and one from Germany (the Max Planck Institute, in the 10^th^ place).

The overall number of publishing institutions grew around 75% between periods (from 18,965 to 33,117). In both periods, around 65% of the institutions published only one paper in HBMS’s top 30 journals. However, at least one of the top 200 institutions was among the authors of 91% and 81% of the papers, in the first and second period, respectively. The network shows a reduction in the total number of clusters (from 12 to 7) between periods. There is also an increase in the number of links between clusters (Figs 1 and 2), showing that the scientific production in HBMS is becoming more unified and revolving around a group of leading organizations from core countries.

### Prevailing HBMS research agenda

**Fig 3:**
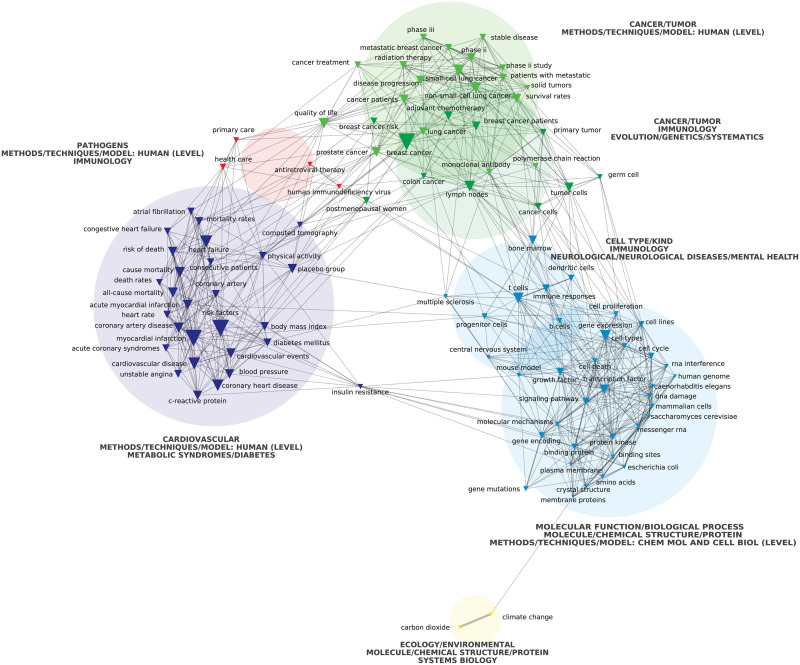
Top 100 HBMS research multi-terms (1999 to 2008) plotted according to co-occurrence using a chi-squared distribution. Source: Authors’ analysis based on WoS data extraction plotted via CorTexT.

**Fig 4:**
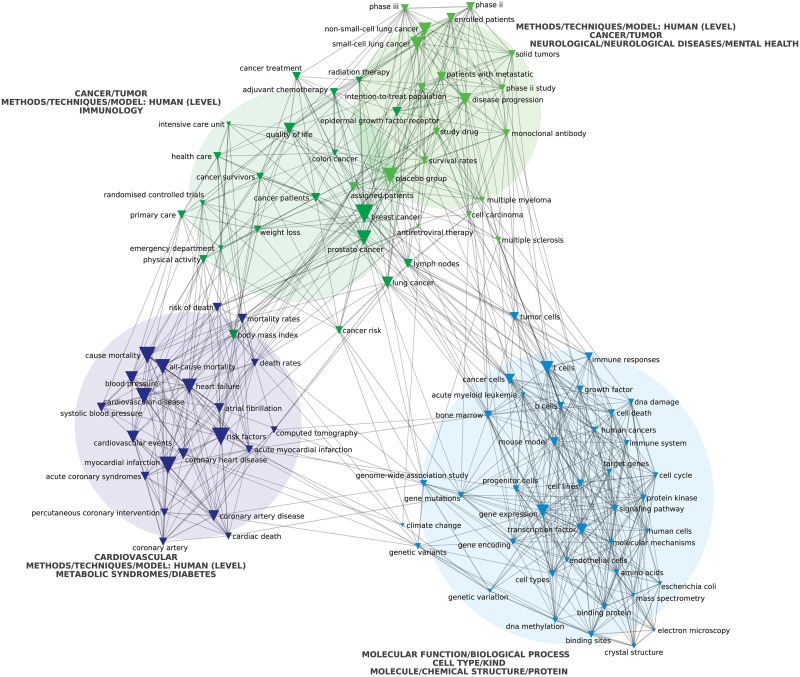
Top 100 HBMS research multi-terms (2009 to 2018) plotted according to co-occurrence using a chi-squared distribution. Source: Authors’ analysis based on WoS data extraction plotted via CorTexT.

Next, we assessed the most prevalent terms found in titles, keywords, and abstracts in this corpus of scientific publications. In both periods, as much as 40% of the most frequent multi-terms derived from the analysed publications are related to general categories linked with molecular and cellular levels such as “protein kinase”, “monoclonal antibody”, “RNA interference”, or “epidermal growth factor receptor” (S3 Table). We also found that terms linked to cancer research, such as “cancer cells” and “tumor growth”, were preponderant (Figs 3 and 4, green circles). Noteworthy, six of the 30 journals with the highest impact factor within the field of HBMS are focused on oncology. Consistently, nearly 15% of the most frequent multi-terms in different documents were classified as related to cancer (S3 Table). In the second period, the predominance of cancer multi-terms increases, and they split into two separate clusters (Fig 4, green circles).

The second most prevalent general category related to a specific group of human diseases is cardiovascular. Both periods present a cluster dominated by multi-terms associated with this general category (Figs 3 and 4, lilac circle), as evidenced by the emergence of terms like “cardiac death” and “coronary heart disease”. In total, 8% of the most frequent multi-terms are associated with this general category (S3 Table).

Other less prevalent multi-terms integrate different clusters associated with neurological diseases and mental health, immunology, metabolic syndromes (such as diabetes), or bone research (Figs 3 and 4). Finally, there are two small and barely connected clusters in the first period associated with environmental terms (yellow circle) and pathogens (salmon circle). In the latter, the Human Immunodeficiency Virus (HIV) is the only mentioned pathogen (Fig 3). Moreover, these clusters disappear entirely in the second period (Fig 4). Consistently, only 1% or less of the most frequent multi-terms in different documents are associated with general categories such as pathogens, microbiology, or environmental (S3 Table).

## Discussion

We presented data supporting the hypothesis that the research agendas of large pharmaceutical firms are intertwined with those of leading academic institutions concentrated in specific countries, which is consistent with the findings of previous studies [15]. Roche, GlaxoSmithKline, Pfizer, Merck, AstraZeneca, and Amgen Inc. participate in the prevailing HBMS knowledge network of organizations, and their influence (number of connections with other institutions of the network) has increased in the last ten years. Given the latter, we can infer that at least part of their research agenda is embedded in the prevailing HBMS research agenda. This allows the possibility of pharmaceutical firms influencing the HBMS research field beyond their direct collaborations with academics. As explained previously, the skewing problem was defined as the tension between academic and commercially oriented research [1,2]. Therefore, our findings provide evidence about the existence of a skewing problem irrespective of direct links with private companies and regardless of researchers’ awareness. Moreover, in line with Rikap [20], large pharmaceutical corporations tend to be directly linked and some of them even belong to the same cluster, thus favouring the thesis of technological cooperation between large pharmaceutical companies in certain steps of their respective innovation networks.

In terms of the geopolitical distribution, we found a predominance of North America (in particular, the US) and Europe. Beyond these regions, Australia, China, Japan, and Israel have between three and seven institutions participating in the prevailing HBMS knowledge network. While there is a majority of US institutions, scientific production in HBMS is becoming more unified and spinning around a group of leading organizations from a core of countries.

Our results suggest that the prevailing HBMS research agenda is dominated by a perspective where medical knowledge is mostly based on research in the field of molecular biology. This claim is supported by the prevalence of terms and topics associated with molecular and cellular biology. Moreover, it prioritizes cancer and cardiovascular research over other pathologies. This prevalence is revealing in terms of the topics that are not part of this agenda. While, by definition, neglected diseases are absent from the agenda, it is significant that research on pathogenic viruses, bacteria or other microorganisms, and biological vectors (for example, bats) is marginal. Although it is difficult to assess, it is conceivable that if these topics would have had a more prevalent place in the high-impact factor journals’ agenda, resulting knowledge would have been a very valuable background to prevent or treat epidemics and pandemics in a more effective way [31]. It is also meaningful to observe that research on prevention, social determinants of health, and assessment of socio-environmental factors influencing disease onset or progression is negligible. Overall, the main focus of the prevailing HBMS agenda appears to be set on therapeutic and specifically pharmacological intervention involving the use of novel drugs or innovative molecular biology techniques. At the same time, prevention and assessment of socio-environmental factors influencing disease onset are almost absent.

As Kaiser described, reductive explanations refer to factors at a lower level than the phenomenon at issue; they concentrate on internal factors and thus ignore or simplify the environment of a system, studying only the parts of an isolated system [32]. In the case of cancer, our results point to a research strategy that ignores the growing evidence of carcinogenicity associated with different environmental pollutants derived from technoscience and related to human activities [33]. While cancer and cardiovascular diseases are among the top ten causes of death worldwide and therefore an enrichment in multi-terms related to research in these disorders was expected, it is noteworthy that other highly prevalent causes of death, including respiratory, diarrhoeal, and infectious diseases are severely underrepresented in the prevailing agenda [34,35]. The fact that our results reveal a growing prevalence of cancer in the prevailing HBMS research agenda should not be surprising. Large pharmaceutical corporations announced a shift towards more profitable diseases such as cancer around ten years ago [36]. As previously reported, the anticancer drugs’ market is highly profitable, even for drugs that represent little or no additional therapeutic value to the population [37–39].

Summing up, one of the main contributions of this article is to approach the skewing dilemma from a novel methodology that enlarges the scope of existing research, overcoming its shared shortfalls. To our knowledge, there is no background of an investigation on the skewing problem that simultaneously provides evidence beyond direct links with private firms and the perceptions of the actors involved. Our contribution addresses all the latter by mapping bibliometric evidence and revealing the network of power relationships that underlies the prevailing HBMS research agenda.

Nonetheless, a significant limitation of our research is that we have only considered one academic research outcome (publications). Our results may end up favoring topics within HBMS that are more often published as papers over other outcomes, such as reports for public authorities, patents, or the creation of a spin-off. Another limitation is that we did not look at the interplay between co-authorship and funding sources. It was shown by previous literature that industry influences HBMS research by sponsoring certain topics and methods [7–9]. Therefore, we may expect both factors to complement each other. More research will be needed to shed light on these aspects. Our future agenda includes this research question.

Additionally, the journal selection process may be considered as another limitation of this study, since it could play a role in creating a bias due to a sampling process focused on a limited set of journals, those with the highest impact factor. Although preliminary data using different sets of high impact journals showed similar results, further research will be necessary to address whether these results can be extrapolated to other journals within the field. In this sense, this work concludes only on what we defined as the prevailing HBMS research agenda, drawn from the 30 journals with the highest impact factor within the field of HBMS. However, it is worth emphasizing that our analysis included some of the most influential interdisciplinary scientific journals and particularly some of the most prestigious journals specialized in microbiology or infectious diseases.

Since we found that certain large pharmaceutical corporations contribute to set HBMS’s prevailing research agenda, further research will also involve comparing the research agendas of these corporations—defined by their scientific publications—with the prevailing HBMS research agenda obtained from our analysis. This will allow us to provide evidence on their level of alignment. Finally, further investigations will also explore the global implications of the lack of diversity, since we found that HBMS’s prevailing research agenda is mainly focused on a few diseases and research topics. A more balanced research agenda, together with epistemological approaches that consider socio-environmental factors associated with disease spreading, could contribute to being better prepared to prevent and treat more diverse pathologies and to improve overall health outcomes.

## Supporting information

S1 Table List of the 30 HBMS journals with the highest impact factors according to WoS. The impact factor (2018), total citations (2018) and total documents (1999–2018) retrieved by WoS for each journal are shown. Selected publications: Science Citation Index Expanded (SCIE), Social Science Citation Index (SSCI). Selected specific categories to retrieve the full list of HBMS journals: ‘Allergy’, ‘Anatomy & Morphology’, ‘Andrology’, ‘Anesthesiology’, ‘Audiology & Speech-Language Pathology’, ‘Biochemical Research Methods’, ‘Biochemistry & Molecular Biology’, ‘Biology’, ‘Biotechnology & Applied Microbiology’, ‘Cardiac & Cardiovascular Systems’, ‘Cell & Tissue Engineering’, ‘Cell Biology’, ‘Chemistry, Medicinal’, ‘Clinical Neurology’, ‘Critical Care Medicine’, ‘Emergency Medicine’, ‘Endocrinology & Metabolism’, ‘Engineering, Biomedical’, ‘Genetics & Heredity’, ‘Health Care Sciences & Services’, ‘Health Policy & Services’, ‘Hematology’, ‘Immunology’, ‘Infectious Diseases’, ‘Integrative & Complementary Medicine’, ‘Medical Ethics’, ‘Medical Informatics’, ‘Medical Laboratory Technology’, ‘Medicine, General & Internal’, ‘Medicine, Legal’, ‘Medicine, Research & Experimental’, ‘Microbiology’, ‘Multidisciplinary Sciences’, ‘Nanoscience & Nanotechnology’, ‘Neuroimaging’, ‘Neurosciences’, ‘Nursing’, ‘Nutrition & Dietetics’, ‘Obstetrics & Gynecology’, ‘Oncology’, ‘Ophthalmology’, ‘Orthopedics’, ‘Otorhinolaryngology’, ‘Parasitology’, ‘Pathology’, ‘Pediatrics’, ‘Peripheral Vascular Disease’, ‘Pharmacology & Pharmacy’, ‘Psychiatry’, ‘Radiology, Nuclear Medicine & Medical Imaging’, ‘Reproductive Biology’, ‘Respiratory System’, ‘Rheumatology’, ‘Social Sciences, Biomedical’, ‘Surgery’, ‘Toxicology’, ‘Transplantation’,’ Tropical Medicine’, ‘Urology & Nephrology’, ‘Virology’.
(PDF)

S2 Table Ranking of scientific institutions and corporations according to their overall publishing frequency in distinct documents for the chosen corpus. Years: 1999–2018. The table displays the top 200 institutions and corporations (red). For each institution or corporation, the number of distinct documents is also shown.
(PDF)

S3 Table Frequency of multi-terms associated to different categories in the prevailing HBMS agenda. The tables display the accumulated frequency of occurrence of multi-terms corresponding to a particular category. The prevailing HBMS research agenda is divided by period.
(PDF)

S1 File HBMS research agenda map (2009–2018)—Alternative corpus. (PDF)

S2 File Original term extraction. (PDF)
