# Correction: Whose shoulders is health research standing on? Determining the key actors and contents of the prevailing biomedical research agenda

**DOI:** 10.1371/journal.pone.0260330

**Published:** 2021-11-16

**Authors:** Federico E. Testoni, Mercedes García Carrillo, Marc-André Gagnon, Cecilia Rikap, Matías Blaustein

The following information is missing from the Funding statement: This study was supported by the Canadian Social Sciences and Humanities Research Council (SSHRC).
