# Correction: Whose shoulders is health research standing on? Determining the key actors and contents of the prevailing biomedical research agenda

**DOI:** 10.1371/journal.pone.0296549

**Published:** 2023-12-28

**Authors:** Federico E. Testoni, Mercedes García Carrillo, Marc-André Gagnon, Cecilia Rikap, Matías Blaustein

There are errors in the Funding statement. The correct Funding statement is as follows: The publication of this work was supported by Carleton University, the Canadian Social Sciences and Humanities Research Council (SSHRC), and the LabEx Institut Francilien de Recherche Innovation Société (IFRIS) exploratory project “The Industry 4.0 and the international division of labour”. Marc-André Gagnon is an Associate Professor at Carleton University. Cecilia Rikap was supported by an IFRIS (www.ifris.org) postdoctoral fellowship during part of the investigation. Matias Blaustein and Cecilia Rikap are members of CONICET. Federico Testoni, and Mercedes García Carrillo were supported by a doctoral fellowship from UBA and a postdoctoral fellowship from CONICET, respectively.

## References

[1] Testoni FE, García Carrillo M, Gagnon M-A, Rikap C, Blaustein M (2021) Whose shoulders is health research standing on? Determining the key actors and contents of the prevailing biomedical research agenda. PLoS ONE 16(4): e0249661. 10.1371/journal.pone.0249661 33826657 PMC8026021

