# Supplementary material for: Whose shoulders is health research standing on? Determining the key actors and contents of the prevailing biomedical research agenda
Source: PLoS One. 2021 Apr 7;16(4):e0249661. doi: 10.1371/journal.pone.0249661 (PMC8026021; doi:10.1371/journal.pone.0249661)
Supplement: S1 Table — The impact factor (2018), total citations (2018) and total documents (1999–2018) retrieved by WoS for each journal are shown. Selected publications: Science Citation Index Expanded (SCIE), Social Science Citation Index (SSCI). Selected specific categories to retrieve the full list of HBMS journals: ‘Allergy’, ‘Anatomy & Morphology’, ‘Andrology’, ‘Anesthesiology’, ‘Audiology & Speech-Language Pathology’, ‘Biochemical Research Methods’, ‘Biochemistry & Molecular Biology’, ‘Biology’, ‘Biotechnology & Applied Microbiology’, ‘Cardiac & Cardiovascular Systems’, ‘Cell & Tissue Engineering’, ‘Cell Biology’, ‘Chemistry, Medicinal’, ‘Clinical Neurology’, ‘Critical Care Medicine’, ‘Emergency Medicine’, ‘Endocrinology & Metabolism’, ‘Engineering, Biomedical’, ‘Genetics & Heredity’, ‘Health Care Sciences & Services’, ‘Health Policy & Services’, ‘Hematology’, ‘Immunology’, ‘Infectious Diseases’, ‘Integrative & Complementary Medicine’, ‘Medical Ethics’, ‘Medical Informatics’, ‘Medical Laboratory Technology’, ‘Medicine, General & Internal’, ‘Medicine, Legal’, ‘Medicine, Research & Experimental’, ‘Microbiology’, ‘Multidisciplinary Sciences’, ‘Nanoscience & Nanotechnology’, ‘Neuroimaging’, ‘Neurosciences’, ‘Nursing’, ‘Nutrition & Dietetics’, ‘Obstetrics & Gynecology’, ‘Oncology’, ‘Ophthalmology’, ‘Orthopedics’, ‘Otorhinolaryngology’, ‘Parasitology’, ‘Pathology’, ‘Pediatrics’, ‘Peripheral Vascular Disease’, ‘Pharmacology & Pharmacy’, ‘Psychiatry’, ‘Radiology, Nuclear Medicine & Medical Imaging’, ‘Reproductive Biology’, ‘Respiratory System’, ‘Rheumatology’, ‘Social Sciences, Biomedical’, ‘Surgery’, ‘Toxicology’, ‘Transplantation’,’ Tropical Medicine’, ‘Urology & Nephrology’, ‘Virology’. (PDF) [file pone.0249661.s001.pdf]

RANK	FULL JOURNAL TITLE	IMPACT FACTOR	TOTAL CITES	TOTAL DOCUMENTS
1	CA-A CANCER JOURNAL FOR CLINICIANS	223.679	32,410	263
2	NEW ENGLAND JOURNAL OF MEDICINE	70.670	344,581	5,969
3	LANCET	59.102	247,292	7,414
4	NATURE REVIEWS DRUG DISCOVERY	57.618	32,266	330
5	NATURE REVIEWS CANCER	51.848	50,529	104
6	JAMA-JOURNAL OF THE AMERICAN MEDICAL ASSOCIATION	51.273	156,350	11,419
7	NATURE REVIEWS IMMUNOLOGY	44.019	41,499	189
8	NATURE REVIEWS GENETICS	43.704	36,697	63
9	NATURE REVIEWS MOLECULAR CELL BIOLOGY	43.351	45,869	87
10	NATURE	43.070	745,692	18,132
11	SCIENCE	41.063	680,994	16,259
12	CELL	36.216	242,829	6,244
13	LANCET ONCOLOGY	35.386	48,822	1,469
14	NATURE REVIEWS MICROBIOLOGY	34.648	29,637	146
15	NATURE REVIEWS CLINICAL ONCOLOGY	34.106	9,626	110
16	WORLD PSYCHIATRY	34.024	5,426	263
17	NATURE NANOTECHNOLOGY	33.407	63,245	1,504
18	NATURE REVIEWS NEUROSCIENCE	33.162	43,107	39
19	NATURE REVIEWS DISEASE PRIMERS	32.274	4,339	174
20	NATURE BIOTECHNOLOGY	31.864	60,971	2,409
21	NATURE MEDICINE	30.641	79,243	3,058
22	LANCET NEUROLOGY	28.755	30,748	668
23	NATURE METHODS	28.467	64,324	1,886
24	JOURNAL OF CLINICAL ONCOLOGY	28.349	154,462	4,840
25	BMJ-BRITISH MEDICAL JOURNAL	27.604	112,901	2,413
26	LANCET INFECTIOUS DISEASES	27.516	23,088	736
27	ANNUAL REVIEW OF BIOCHEMISTRY	26.922	20,344	630
28	CANCER DISCOVERY	26.370	13,715	555
29	NATURE GENETICS	25.455	93,920	4,036
30	EUROPEAN HEART JOURNAL	24.889	58,233	4,636
