# Supplementary material for: Whose shoulders is health research standing on? Determining the key actors and contents of the prevailing biomedical research agenda
Source: PLoS One. 2021 Apr 7;16(4):e0249661. doi: 10.1371/journal.pone.0249661 (PMC8026021; doi:10.1371/journal.pone.0249661)
Supplement: S2 Table — Years: 1999–2018. The table displays the top 200 institutions and corporations (red). For each institution or corporation, the number of distinct documents is also shown. (PDF) [file pone.0249661.s002.pdf]

| RANK | INSTITUTION/CORPORATION | NUMBER OF DISTINCT DOCUMENTS |
|---|---|---|
| 1 | HARVARD | 12067 |
| 2 | UNIV CALIF | 11090 |
| 3 | UNIV LONDON | 5598 |
| 4 | UNIV TEXAS | 4874 |
| 5 | UNIV WASHINGTON | 4801 |
| 6 | UNIV STANFORD | 3624 |
| 7 | NIH | 3462 |
| 8 | JOHNS HOPKINS UNIV | 3256 |
| 9 | UNIV OXFORD | 2968 |
| 10 | MAX PLANCK | 2831 |
| 11 | MIT | 2600 |
| 12 | UNIV COLUMBIA | 2529 |
| 13 | UNIV TORONTO | 2474 |
| 14 | UNIV CAMBRIDGE | 2432 |
| 15 | DUKE UNIV | 2419 |
| 16 | UNIV PENN | 2377 |
| 17 | MEM SLOAN KETTERING CANC CTR | 2300 |
| 18 | UNIV YALE | 2194 |
| 19 | UNIV MICHIGAN | 2171 |
| 20 | UNIV CORNELL | 2043 |
| 21 | MAYO CLIN | 1889 |
| 22 | UNIV COLORADO | 1733 |
| 23 | MT SINAI HEALTH SYSTEM | 1718 |
| 24 | UNIV COPENHAGEN | 1705 |
| 25 | UNIV CHICAGO | 1611 |
| 26 | UNIV N CAROLINA | 1610 |
| 27 | KAROLINSKA INST | 1600 |
| 28 | UNIV WISCONSIN | 1507 |
| 29 | CALTECH | 1441 |
| 30 | UNIV NORTHWESTERN | 1429 |
| 31 | UNIV ERASMUS MC | 1409 |
| 32 | UNIV PITTSBURGH | 1392 |
| 33 | UNIV TOKYO | 1380 |
| 34 | HARVARD & MIT | 1358 |
| 35 | UNIV EMORY | 1285 |
| 36 | BAYLOR COLL MED | 1267 |
| 37 | UNIV NEW YORK | 1265 |
| 38 | SORBONNE UNIV | 1205 |
| 39 | UNIV MINNESOTA | 1198 |
| 40 | UNIV EDINBURGH | 1195 |
| 41 | UNIV UTRECHT | 1157 |
| 42 | UNIV AMSTERDAM | 1149 |
| 43 | CNRS | 1148 |
| 44 | UNIV MARYLAND | 1140 |
| 45 | UNIV BRITISH COLUMBIA | 1125 |
| 46 | UNIV MUNICH | 1123 |
| 47 | UNIV ZURICH | 1112 |
| 48 | UNIV MELBOURNE | 1098 |
| 49 | UNIV VANDERBILT | 1079 |
| 50 | UNIV S CALIF | 1078 |
| 51 | UNIV LEIDEN | 1078 |
| 52 | UNIV MCGILL | 1066 |
| 53 | UNIV BOSTON | 1063 |
| 54 | UNIV HEIDELBERG | 1059 |
| 55 | HOWARD HUGHES MED INST | 1049 |
| 56 | UNIV OHIO STATE | 1049 |
| 57 | UNIV STATE NEW YORK | 1040 |
| 58 | UNIV SYDNEY | 1034 |
| 59 | HELMHOLTZ ASSOC GERMAN RES CTR | 1017 |
| 60 | INSERM | 1010 |
| 61 | CHINESE ACAD SCI | 1003 |
| 62 | FRED HUTCHINSON CANC RES CTR | 1002 |
| 63 | UNIV PARIS DESCARTES | 985 |
| 64 | WELLCOME TRUST | 981 |
| 65 | UNIV ALABAMA | 980 |
| 66 | CLIN CLEVELAND | 976 |
| 67 | UNIV GRONINGEN | 949 |
| 68 | UNIV MANCHESTER | 936 |
| 69 | UNIV HELSINKI | 932 |
| 70 | KU LEUVEN | 929 |

| | | |
|---|---|---|
| 71 | UNIV RABOUD NIJMEGEN | 913 |
| 72 | UNIV MASSACHUSETTS | 899 |
| 73 | UNIV ROCKEFELLER | 893 |
| 74 | UNIV BRISTOL | 892 |
| 75 | SCRIPPS RES INST | 890 |
| 76 | UNIV UPPSALA | 877 |
| 77 | UNIV UTAH | 875 |
| 78 | UNIV ARIZONA | 860 |
| 79 | UNIV FREE AMSTERDAM | 860 |
| 80 | UNIV OREGON | 857 |
| 81 | UNIV MCMASTER | 854 |
| 82 | US DEPT OF ENERGY | 851 |
| 83 | MED RES COUNCIL UK | 837 |
| 84 | UNIV ILLINOIS | 837 |
| 85 | UNIV INDIANA | 835 |
| 86 | UNIV LEEDS | 823 |
| 87 | ROCHE | 820 |
| 88 | UNIV PRINCETON | 816 |
| 89 | UNIV GLASGOW | 810 |
| 90 | UNIV KYOTO | 796 |
| 91 | UNIV AARHUS | 787 |
| 92 | UNIV HOSP | 787 |
| 93 | UNIV VIENNA | 782 |
| 94 | CDC | 771 |
| 95 | UNIV PARIS DIDEROT | 770 |
| 96 | UNIV TECH MUNICH | 768 |
| 97 | UNIV QUEENSLAND | 756 |
| 98 | CHILDRENS HOSP | 755 |
| 99 | UNIV PARIS SACLAY | 747 |
| 100 | UNIV BASEL | 734 |
| 101 | NASA | 728 |
| 102 | UNIV BIRMINGHAM | 723 |
| 103 | UNIV NEWCASTLE | 723 |
| 104 | RIKEN | 721 |
| 105 | UNIV OSLO | 721 |
| 106 | HOSP ST JUDE CHILDRENS RES | 718 |
| 107 | UNIV BARCELONA | 710 |
| 108 | UNIV PENN STATE | 707 |
| 109 | LONDON SCH HYG & TROP MED | 704 |
| 110 | UNIV ALBERTA | 702 |
| 111 | UNIV BERN | 699 |
| 112 | HOSP UNIV CHARITE | 696 |
| 113 | UNIV LUND | 693 |
| 114 | UNIV MONTREAL | 691 |
| 115 | UNIV IOWA | 688 |
| 116 | EMBL | 686 |
| 117 | UNIV ROCHESTER | 672 |
| 118 | UNIV LYON | 668 |
| 119 | UNIV VIRGINIA | 668 |
| 120 | UNIV BROWN | 661 |
| 121 | UNIV CASE WESTERN RESERVE | 661 |
| 122 | UNIV FLORIDA | 654 |
| 123 | UNIV MONASH | 646 |
| 124 | UNIV HAMBURG | 636 |
| 125 | NOVARTIS | 633 |
| 126 | SWISS FED INST TECHNOL EPFL | 630 |
| 127 | INST GUSTAVE ROUSSY | 630 |
| 128 | ETH ZURICH | 627 |
| 129 | UNIV TUBINGEN | 625 |
| 130 | UNIV FREIBURG | 622 |
| 131 | WHO | 612 |
| 132 | INST PASTEUR | 610 |
| 133 | UNIV GENEVA | 605 |
| 134 | UNIV LIVERPOOL | 597 |
| 135 | UNIV SOUTHAMPTON | 596 |
| 136 | JAPAN SCI & TECHNOL AGCY | 581 |
| 137 | HOSP ROYAL MARSDEN | 581 |
| 138 | UNIV LAUSANNE | 579 |
| 139 | UNIV YESHIVA | 575 |
| 140 | UNIV MIAMI | 574 |
| 141 | UNIV COLOGNE | 573 |

| | | |
|---|---|---|
| 142 | UNIV LEICESTER | 571 |
| 143 | UNIV MILANO | 567 |
| 144 | UNIV PEKING | 565 |
| 145 | UNIV TUFTS | 557 |
| 146 | IMPERIAL COLL LONDON | 549 |
| 147 | UNIV OSAKA | 531 |
| 148 | UNIV NATL SINGAPORE | 529 |
| 149 | GERMAN CANC RES CTR | 527 |
| 150 | UNIV TENNESSEE | 526 |
| 151 | INST CANC RES | 526 |
| 152 | UNIV WAKE FOREST | 524 |
| 153 | UNIV CALGARY | 524 |
| 154 | UNIV SEOUL NATL | 524 |
| 155 | UNIV RUTGERS | 513 |
| 156 | UNIV BONN | 509 |
| 157 | UNIV NEW SOUTH WALES | 506 |
| 158 | UNIV TEL AVIV | 505 |
| 159 | UNIV NOTTINGHAM | 503 |
| 160 | UNIV MUNSTER | 501 |
| 161 | GLAXOSMITHKLINE | 496 |
| 162 | UNIV DUISBURG ESSEN | 491 |
| 163 | WEIZMANN INST SCI | 491 |
| 164 | UNIV ULM | 490 |
| 165 | UNIV HAWAII | 483 |
| 166 | UNIV OTTAWA | 480 |
| 167 | HOSP SICK CHILDREN | 480 |
| 168 | UNIV LILLE | 479 |
| 169 | UNIV W AUSTRALIA | 478 |
| 170 | UNIV HEBREW HADASSAH | 464 |
| 171 | UNIV GOTHENBURG | 463 |
| 172 | UNIV PADUA | 461 |
| 173 | UNIV GHENT | 461 |
| 174 | HOSP CHILDRENS PHILADELPHIA | 459 |
| 175 | UNIV SHEFFIELD | 454 |
| 176 | SALK INST BIOL STUDIES | 454 |
| 177 | CSIC | 452 |
| 178 | UNIV BORDEAUX | 450 |
| 179 | KU LOUVAIN | 450 |
| 180 | UNIV HANNOVER | 448 |
| 181 | UNIV ROMA LA SAPIENZA | 447 |
| 182 | UNIV TORINO | 444 |
| 183 | PFIZER | 435 |
| 184 | UNIV WURZBURG | 434 |
| 185 | UNIV MAASTRICHT | 432 |
| 186 | UNIV BERGEN | 429 |
| 187 | KAISER PERMANENTE ORG | 427 |
| 188 | NETHERLANDS CANC INST | 423 |
| 189 | AMGEN INC | 420 |
| 190 | CONSIGLIO NAZL RIC CNR | 419 |
| 191 | UNIV CARDIFF | 416 |
| 192 | UNIV GOTTINGEN | 414 |
| 193 | HOSP PRINCESS MARGARET | 413 |
| 194 | UNIV MONTPELLIER | 411 |
| 195 | UNIV FREE BRUXELLES | 411 |
| 196 | COLD SPRING HARBOR LAB | 409 |
| 197 | UNIV S CAROLINA | 405 |
| 198 | MERCK | 402 |
| 199 | UNIV GEORGETOWN | 400 |
| 200 | UNIV AUSTRALIAN NATL | 399 |
