# Supplementary material for: Whose shoulders is health research standing on? Determining the key actors and contents of the prevailing biomedical research agenda
Source: PLoS One. 2021 Apr 7;16(4):e0249661. doi: 10.1371/journal.pone.0249661 (PMC8026021; doi:10.1371/journal.pone.0249661)
Supplement: S3 Table — The tables display the accumulated frequency of occurrence of multi-terms corresponding to a particular category. The prevailing HBMS research agenda is divided by period. (PDF) [file pone.0249661.s003.pdf]

GLOBAL 1999-2008 (TOP JOURNALS)		
CATEGORY	NUMBER OF DISTINCT DOCUMENTS	PERCENTAGE
METHODS/TECHNIQUES/MODEL: HUMAN (LEVEL)	9768	19,1
MOLECULAR FUNCTION/BIOLOGICAL PROCCESS	8002	15,6
CANCER/TUMOR	6831	13,4
MOLECULE/CHEMICAL STRUCTURE/PROTEIN	5472	10,7
CARDIOVASCULAR	4282	8,4
METHODS/TECHNIQUES/MODEL: CHEM MOL AND CELL BIOL (LEVEL)	3858	7,5
CELL TYPE/KIND	3298	6,4
IMMUNOLOGY	1704	3,3
METHODS/TECHNIQUES/MODEL: ANIMAL (LEVEL)	1589	3,1
EVOLUTION/GENETICS/SYSTEMATICS	1533	3,0
MICROBIOLOGY/APPLIED MICROBIOLOGY	855	1,7
CELLULAR COMPONENT	797	1,6
NEUROLOGICAL/NEUROLOGICAL DISEASES/MENTAL HEALTH	766	1,5
PATHOGENS	651	1,3
METABOLIC SYNDROMES/DIABETES	572	1,1
BONE	533	1,0
ECOLOGY/ENVIRONMENTAL	317	0,6
METHODS/TECHNIQUES/MODEL: PLANT (LEVEL)	204	0,4
SYSTEMS BIOLOGY	124	0,2
TOTAL	51156	100,0

GLOBAL 2009-2018 (TOP JOURNALS)		
CATEGORY	NUMBER OF DISTINCT DOCUMENTS	PERCENTAGE
METHODS/TECHNIQUES/MODEL: HUMAN (LEVEL)	11524	19,5
MOLECULAR FUNCTION/BIOLOGICAL PROCCESS	9210	15,6
CANCER/TUMOR	8370	14,2
MOLECULE/CHEMICAL STRUCTURE/PROTEIN	5488	9,3
METHODS/TECHNIQUES/MODEL: CHEM MOL AND CELL BIOL (LEVEL)	4976	8,4
CARDIOVASCULAR	4502	7,6
CELL TYPE/KIND	3997	6,8
EVOLUTION/GENETICS/SYSTEMATICS	2463	4,2
IMMUNOLOGY	2020	3,4
METHODS/TECHNIQUES/MODEL: ANIMAL (LEVEL)	1885	3,2
NEUROLOGICAL/NEUROLOGICAL DISEASES/MENTAL HEALTH	829	1,4
MICROBIOLOGY/APPLIED MICROBIOLOGY	751	1,3
CELLULAR COMPONENT	737	1,2
PATHOGENS	603	1,0
BONE	519	0,9
METABOLIC SYNDROMES/DIABETES	504	0,9
ECOLOGY/ENVIRONMENTAL	335	0,6
SYSTEMS BIOLOGY	204	0,3
METHODS/TECHNIQUES/MODEL: PLANT (LEVEL)	169	0,3
TOTAL	59086	100,0
