# Supplementary material for: Whose shoulders is health research standing on? Determining the key actors and contents of the prevailing biomedical research agenda
Source: PLoS One. 2021 Apr 7;16(4):e0249661. doi: 10.1371/journal.pone.0249661 (PMC8026021; doi:10.1371/journal.pone.0249661)
Supplement: S1 File — (PDF) [file pone.0249661.s004.pdf]

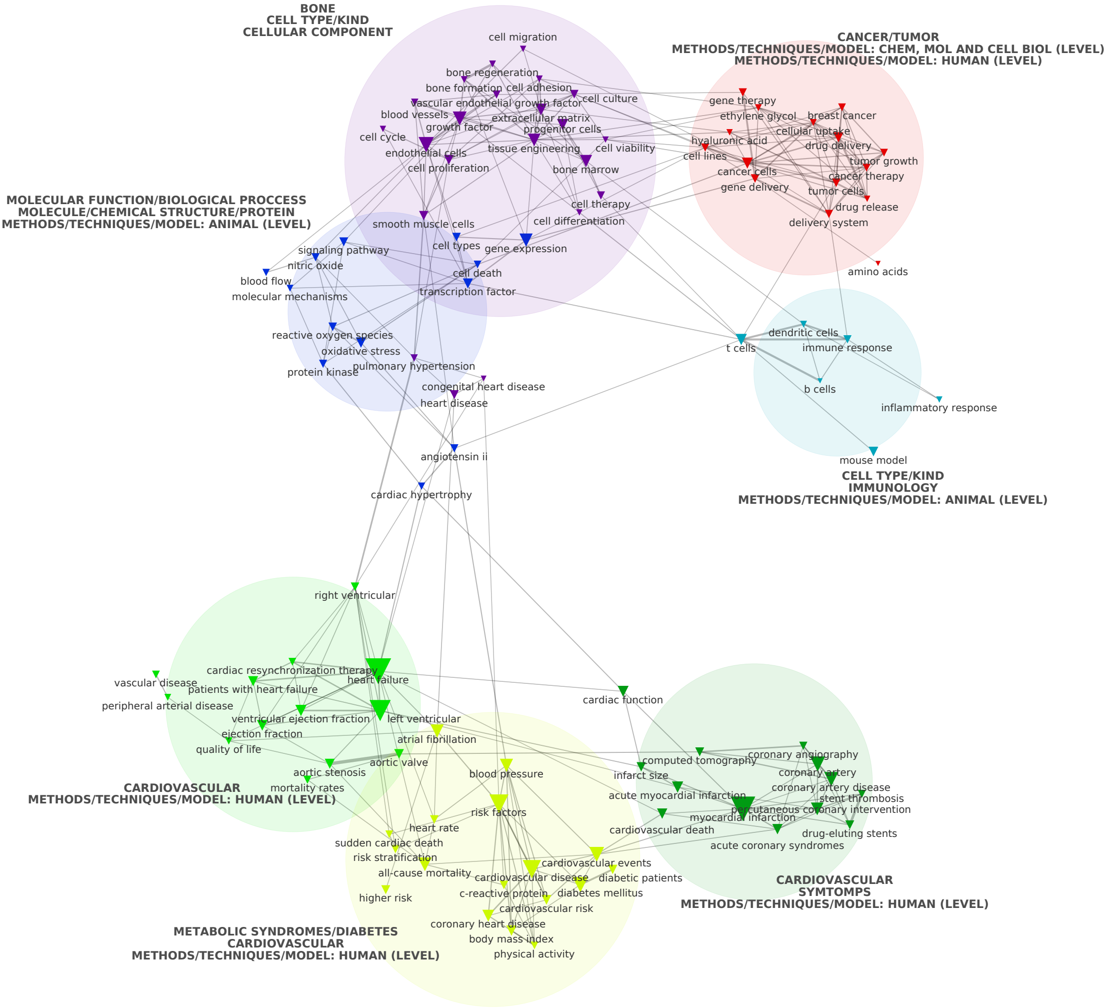

Alternative corpus analysis: Top HBMS research multi-terms (2009 to 2018) plotted according to co-occurrence using a chi-squared distribution. Source: alternative author´s analysis, based on WoS data extraction plotted via CorTexT. Journals included in the alternative corpus: 1: BIOLOGICAL REVIEWS, BIOMATERIALS, BMJ-BRITISH MEDICAL JOURNAL, CA-A CANCER breast JOURNAL FOR CLINICIANS, CELL, CIRCULATION, CIRCULATION RESEARCH, EUROPEAN HEART JOURNAL, JOURNAL OF THE AMERICAN COLLEGE OF CARDIOLOGY, LANCET DIABETES & ENDOCRINOLOGY, LANCET GLOBAL HEALTH, LANCET HIV, LANCET INFECTIOUS DISEASES, LANCET NEUROLOGY, NATURE BIOTECHNOLOGY, NATURE CELL BIOLOGY, NATURE MEDICINE, NATURE NEUROSCIENCE, NATURE REVIEWS CANCER, NATURE REVIEWS DRUG DISCOVERY, NATURE REVIEWS ENDOCRINOLOGY, NATURE REVIEWS IMMUNOLOGY, NATURE REVIEWS MICROBIOLOGY, NATURE REVIEWS MOLECULAR CELL BIOLOGY, NATURE REVIEWS NEUROLOGY, NATURE REVIEWS NEUROSCIENCE, SCIENCE.
