# Supplementary material for: Whose shoulders is health research standing on? Determining the key actors and contents of the prevailing biomedical research agenda
Source: PLoS One. 2021 Apr 7;16(4):e0249661. doi: 10.1371/journal.pone.0249661 (PMC8026021; doi:10.1371/journal.pone.0249661)
Supplement: S2 File — (PDF) [file pone.0249661.s005.pdf]

Stem	Main form	Forms	n	C-value	Gfidf	Specificity chi2	Occurrences	Cooccurrences
30-day mortal	30-day mortality	30-day mortality	2	511.4327973	1.775956284	1808.995139	183	2466
absolut differ	absolute difference	absolute difference	2	456.3554192	1.33640553	1177.067513	225	2911
acid amino	amino acids	amino acids|&|amino acid	2	854.4861814	1.212053571	2810.390228	448	1777
activ antitumor	antitumor activity	antitumor activity	2	704.9904406	1.172774869	6436.446637	382	5468
activ kinas	kinase activity	kinase activity|&|activity of the kinase	2	428.0299104	1.119341564	3541.383915	243	1554
activ physic	physical activity	physical activity	2	1021.291955	1.739946381	3518.714405	373	4348
activ site	active site	active site|&|active sites	2	837.1761482	1.245901639	21522.75596	427	1731
activ transcript	transcriptional activation	transcriptional activation|&|transcriptional activity	2	546.0528636	1.087774295	5678.469058	319	1709
acut coronari patient syndrom	patients with acute coronary syndromes	patients with acute coronary syndromes|&|patients with acute coronary syndrome|&|acute coronary syndrome patients|&|patients with an acute coronary syndrome|&|acute coronary syndromes in patients|&|acute coronary syndrome in patients|&|p	5	415.0137679	1.142076503	8916.926671	183	2273
acut coronari syndrom	acute coronary syndromes	acute coronary syndromes|&|acute coronary syndrome	3	1799.054898	1.143939394	3333.231888	912	9497
acut infarct myocardi	acute myocardial infarction	acute myocardial infarction	3	2388.811305	1.309031556	3068.726215	1036	10257
acut infarct myocardi patient	patients with acute myocardial infarction	patients with acute myocardial infarction|&|acute myocardial infarction in patients|&|patients with an acute myocardial infarction|&|acute myocardial infarction patients|&|patients after acute myocardial infarction|&|patients without acute myocardial i	5	413.028056	1.155555556	9178.94607	180	2109
acut leukemia lymphoblast	acute lymphoblastic leukemia	acute lymphoblastic leukemia|&|Acute Lymphoblastic Leukemia|&|Lymphoblastic Acute Leukemia	3	921.3702789	1.045045045	1553.866988	531	4402
acut leukemia myeloid	acute myeloid leukemia	acute myeloid leukemia|&|Acute Myeloid Leukemia	3	1231.141321	1.024793388	40621.73983	605	6108
adapt immun	adaptive immunity	adaptive immunity|&|adaptive immune	2	513.0064367	1.143859649	13802.59288	364	2218
adenocarcinoma lung	lung adenocarcinoma	lung adenocarcinoma|&|lung adenocarcinomas|&|adenocarcinoma of the lung|&|adenocarcinomas of the lung|&|adenocarcinoma of lung	2	454.7817798	1.710059172	1897.545914	169	1634
adjust hazard ratio	adjusted hazard ratio	adjusted hazard ratio|&|adjusted hazard ratios	3	1356.241165	1.171526587	3655.751979	583	9060
adjust hr	adjusted HR	adjusted HR	2	401.278041	1.363636364	2422.875069	187	3039
adjust odd ratio	adjusted odds ratio	adjusted odds ratio	3	1175.54139	1.145067698	7863.866798	517	6376
adjuv chemotherapi	adjuvant chemotherapy	adjuvant chemotherapy|&|Adjuvant chemotherapy|&|Adjuvant Chemotherapy|&|chemotherapy in the adjuvant|&|Chemotherapy as Adjuvant	2	1647.600427	1.546528804	2216.286132	677	8459
adjuv therapi	adjuvant therapy	adjuvant therapy	2	708.1377194	1.282051282	1739.939305	362	4680
adjuv treatment	adjuvant treatment	adjuvant treatment|&|treatment in the adjuvant	2	557.0683392	1.195945946	1436.223407	296	3850
advers common event	common adverse events	common adverse events	3	532.1707645	1.011320755	1975.321603	265	4190
advers event	adverse events	adverse events|&|Adverse events|&|adverse event|&|events adverse	2	5097.01794	1.271692187	9186.426834	2765	39060
advers event seriou	serious adverse events	serious adverse events|&|Serious adverse events|&|serious adverse event	3	2362.997051	1.119473189	5996.003576	1063	14932
age children	children aged	children aged|&|aged children	2	597.982963	1.279461279	1270.974188	297	2562
age gestat	gestational age	gestational age	2	539.7583061	1.566210046	1300.102893	219	2191
age median	median age	median age|&|Median age	2	1795.522528	1.03164557	2820.190171	1106	15165
age patient	patients aged	patients aged|&|patient age|&|patients age|&|Patients aged|&|age of the patients|&|aged patients|&|age of the patient|&|age of patients|&|patients of any age|&|age in patients|&|patients below the age|&|patients under the age|&|patients on age|&|p	2	1686.941411	1.105154639	1161.805622	970	13459
age women	women aged	women aged|&|women age|&|aged women|&|women under the age|&|women over the age|&|age in women|&|women across age|&|women at the age|&|women and age|&|women between the age|&|women by age|&|age among women	2	1048.043825	1.273422562	1962.365083	523	6493
aim studi	aim of this study	aim of this study|&|aim of the study|&|study aim|&|aim in this study|&|study with the aim|&|aim this study	4	867.0752964	1	1935.421759	551	6066
alcohol consumpt	alcohol consumption	alcohol consumption|&|consumption of alcohol	2	481.5336492	1.709497207	1473.225834	179	2097
all-caus mortal	all-cause mortality	all-cause mortality|&|All-cause mortality	2	2198.374209	1.64159812	2901.942254	851	12181
alloc treatment	treatment allocation	treatment allocation	2	530.3164698	1.024316109	3072.024386	329	4684
american cancer societi	American Cancer Society	American Cancer Society|&|Cancer From the American Society	3	536.1421881	1.210762332	1359.399553	223	2186
american clinic oncolog societi	American Society of Clinical Oncology	American Society of Clinical Oncology|&|American Society for Clinical Oncology|&|Clinical Oncology and American Society	5	10150.95876	1.016302187	43504.27596	5030	64751
analysi intention-to-treat	intention-to-treat analysis	intention-to-treat analysis	2	586.9674874	1.068767908	1382.217369	349	4424
analysi interim	interim analysis	interim analysis	2	484.6809279	1.171102662	1170.966455	263	3835
analysi multivari	multivariate analysis	multivariate analysis|&|multivariable analysis|&|Multivariate analysis|&|Multivariable analysis	2	2549.29579	1.084337349	5913.312063	1494	19369
analysi primari	primary analysis	primary analysis	2	561.7892574	1.108695652	1265.985986	322	4617
analysi regress	regression analysis	regression analysis	2	484.6809279	1.01650165	3117.206485	619	8193
angina unstabl	unstable angina	unstable angina	2	604.2775205	1.342657343	2327.713625	286	3889
angiographi coronari	coronary angiography	coronary angiography	2	535.037388	1.259259259	3040.422326	270	2804
angstrom resolut	angstrom resolution	angstrom resolution|&|Angstrom resolution	2	1026.012873	1.028391167	37624.19872	634	2709
antibodi monoclon	monoclonal antibody	monoclonal antibody|&|monoclonal antibodies	2	1137.741269	1.105504587	1726.4466	654	7031
antibodi respons	antibody responses	antibody responses|&|antibody response	2	421.7353529	1.307317073	2709.633544	205	1421
antigen prostate-specif	prostate-specific antigen	prostate-specific antigen	2	709.7113587	1.036781609	9230.909396	435	5285
antiretrovir therapi	antiretroviral therapy	antiretroviral therapy	2	786.8196882	1.404494382	3414.671463	357	3318
apoptot cell	apoptotic cells	apoptotic cells|&|apoptotic cell	2	407.5725985	1.692810458	6657.187766	153	898
arabidopsi thaliana	Arabidopsis thaliana	Arabidopsis thaliana	2	689.2540469	1.00228833	2963.845777	437	1665
arm b	arm B	arm B|&|B arm	2	634.1766687	2.779310345	1639.907475	145	2140
arm treatment	treatment arms	treatment arms|&|treatment arm|&|treatment in both arms|&|arm after treatment|&|arm A treatment|&|treatment in each arm|&|treatment on both arms	2	582.2465693	1.241610738	1638.253089	298	4202
arteri coronari	coronary artery	coronary artery|&|coronary arteries|&|artery coronary	2	878.090772	1.17721519	3625.220307	489	5001
arteri coronari diseas	coronary artery disease	coronary artery disease	3	2148.540176	1.192943771	36989.5011	1027	11052
arteri diseas	artery disease	artery disease	2	618.4402749	1.267741935	37935.44053	1400	14840
assess patient	assessable patients	assessable patients|&|patients assessable	2	549.2001424	1.122186495	4029.289483	311	4344
assign patient	assigned patients	assigned patients|&|patients assigned	2	777.3778519	1.066954644	1921.3192	463	6508
assign treatment	treatment assignment	treatment assignment|&|assigned treatment	2	552.3474211	1.038461538	3215.118125	338	4911
associ genome-wid studi	genome-wide association study	genome-wide association study|&|genome-wide association studies|&|Genome-wide association study|&|Genome-wide association studies	3	2023.440332	1.040858018	137827.5081	979	8162
associ signific	significant association	significant association|&|significant associations	2	745.9050644	1.079726651	2209.01198	439	5087
associ studi	association study	association study|&|association studies|&|study the association|&|study of the association|&|study of association	2	505.1382398	1.110726644	125180.5808	1246	10007
atlant north	North Atlantic	North Atlantic	2	585.393848	1.367647059	6610.261866	322	1350
atrial fibril	atrial fibrillation	atrial fibrillation|&|Atrial fibrillation	2	2281.777096	1.410505837	1540.89044	1128	10281
atrial fibril patient	patients with atrial fibrillation	patients with atrial fibrillation|&|atrial fibrillation patients|&|atrial fibrillation in patients|&|patients without atrial fibrillation|&|patients in atrial fibrillation|&|atrial fibrillation in some patients|&|atrial patients fibrillation	4	476.5708339	1.318681319	8424.1287	182	1995
autoimmun diseas	autoimmune disease	autoimmune disease|&|autoimmune diseases	2	535.037388	1.188811189	3462.485798	286	1888
autolog stem-cel transplant	autologous stem-cell transplantation	autologous stem-cell transplantation	3	418.9851915	1.159340659	11698.94005	182	2385
b cell	B cells	B cells|&|B cell	2	1756.181544	1.746478873	5258.11272	639	3733
b nf-kappa	NF-kappa B	NF-kappa B	2	741.1841463	1.635416667	2926.706718	323	2258
base pair	base pairs	base pairs|&|base pair	2	539.7583061	1.166666667	3642.634546	294	1207
basi genet	genetic basis	genetic basis	2	446.9135829	1.04029304	1896.651022	273	1521
basi molecular	molecular basis	molecular basis	2	804.1297213	1.013888889	3200.533986	504	2300
basi structur	Structural basis	Structural basis|&|structural basis|&|Structural Basis	2	914.2844777	1.015734266	3995.855816	572	2954
b-cell lymphoma	B-cell lymphoma	B-cell lymphoma|&|B-Cell Lymphoma|&|B-cell lymphomas|&|lymphoma and B-cell	2	520.8746336	1.161403509	1138.831988	418	3931
beneficiari medicar	Medicare beneficiaries	Medicare beneficiaries	2	415.4407954	1.534883721	1437.894878	172	1976
bind protein	binding protein	binding protein|&|binding proteins|&|protein binding	2	505.1382398	1.035483871	2770.699151	310	1456
bind site	binding sites	binding sites|&|binding site	2	1054.338382	1.107438017	8679.383621	605	2895
biolog system	systems biology	systems biology|&|biological systems	2	418.5880741	1.055555556	1441.24808	252	830
black hole	black holes	black holes|&|black hole	2	731.74231	1.890243902	2206.328887	246	751
block size	block size	block size|&|block sizes	2	405.9989591	1.0078125	3364.744122	256	3881
blood diastol pressur	diastolic blood pressure	diastolic blood pressure	3	446.7851568	1.315789474	4502.994576	171	2546
blood peripher	peripheral blood	peripheral blood	2	505.1382398	1.206766917	1487.988251	266	2641

<table>
<tr><th></th><th></th><th></th><th></th><th></th><th></th><th></th><th></th><th></th></tr>
<tr><td>blood pressur</td><td>blood pressure</td><td>blood pressure|&|Blood pressure</td><td>2</td><td>2851.43455</td><td>1.653284672</td><td>4452.426003</td><td>1415</td><td>16380</td></tr>
<tr><td>blood pressur systol</td><td>systolic blood pressure</td><td>systolic blood pressure</td><td>3</td><td>1195.398508</td><td>1.436754177</td><td>10934.79322</td><td>419</td><td>6227</td></tr>
<tr><td>bodi index mass</td><td>body mass index</td><td>body mass index</td><td>3</td><td>2005.568926</td><td>1.175785797</td><td>30633.46784</td><td>859</td><td>11935</td></tr>
<tr><td>bodi kilogram weight</td><td>kilogram of body weight</td><td>kilogram of body weight</td><td>4</td><td>424.9423269</td><td>1.004694836</td><td>16458.4345</td><td>213</td><td>2712</td></tr>
<tr><td>bodi weight</td><td>body weight</td><td>body weight|&|weight and body|&|weight of the body</td><td>2</td><td>739.6105069</td><td>1.323943662</td><td>1506.800715</td><td>566</td><td>6507</td></tr>
<tr><td>body-mass index</td><td>body-mass index</td><td>body-mass index</td><td>2</td><td>536.6110273</td><td>1.171821306</td><td>1284.692077</td><td>291</td><td>3207</td></tr>
<tr><td>bone marrow</td><td>bone marrow</td><td>bone marrow|&|Bone marrow|&|bone and marrow|&|bone without marrow</td><td>2</td><td>2097.661289</td><td>1.395811518</td><td>3629.687207</td><td>1123</td><td>10108</td></tr>
<tr><td>brain metastas</td><td>brain metastases</td><td>brain metastases</td><td>2</td><td>509.8591579</td><td>2.117647059</td><td>1188.12116</td><td>153</td><td>2051</td></tr>
<tr><td>break dna double-strand</td><td>DNA double-strand breaks</td><td>DNA double-strand breaks|&|double-strand DNA breaks|&|double-strand breaks in DNA|&|DNA via double-strand breaks</td><td>3</td><td>399.1280734</td><td>1.036082474</td><td>4444.531731</td><td>194</td><td>1175</td></tr>
<tr><td>breast cancer</td><td>breast cancer</td><td>breast cancer|&|Breast Cancer|&|breast cancers|&|Breast cancer|&|cancers of the breast|&|Cancer Breast|&|cancer in the breast|&|cancer of the breast</td><td>2</td><td>9575.595605</td><td>1.568703274</td><td>13142.24251</td><td>5347</td><td>52589</td></tr>
<tr><td>breast cancer early-stag</td><td>early-stage breast cancer</td><td>early-stage breast cancer</td><td>3</td><td>623.5135077</td><td>1.308333333</td><td>2741.526075</td><td>240</td><td>2975</td></tr>
<tr><td>breast cancer invas</td><td>invasive breast cancer</td><td>invasive breast cancer|&|invasive cancer in the breast|&|invasive cancer of the breast|&|breast with invasive cancer|&|invasive cancer in breast</td><td>3</td><td>474.5851221</td><td>1.278074866</td><td>1999.991753</td><td>187</td><td>2528</td></tr>
<tr><td>breast cancer metastat</td><td>metastatic breast cancer</td><td>metastatic breast cancer|&|Metastatic Breast Cancer|&|breast cancer metastatic</td><td>3</td><td>1268.869845</td><td>1.298780488</td><td>6556.031798</td><td>595</td><td>7570</td></tr>
<tr><td>breast cancer patient</td><td>breast cancer patients</td><td>breast cancer patients|&|patients with breast cancer|&|breast cancer in patients|&|breast cancer for patients|&|breast cancer and patients|&|breast cancer between patients</td><td>3</td><td>1395.955401</td><td>1.309124767</td><td>11682.75247</td><td>556</td><td>6966</td></tr>
<tr><td>breast cancer risk</td><td>breast cancer risk</td><td>breast cancer risk|&|risk of breast cancer|&|risk for breast cancer|&|risk breast cancer|&|breast cancer and risk</td><td>3</td><td>724.7848099</td><td>1.477732794</td><td>10257.37721</td><td>254</td><td>3143</td></tr>
<tr><td>c degre</td><td>degrees C</td><td>degrees C</td><td>2</td><td>920.5790352</td><td>1.280087527</td><td>2716.670423</td><td>457</td><td>1865</td></tr>
<tr><td>caenorhabd elegan</td><td>Caenorhabditis elegans</td><td>Caenorhabditis elegans</td><td>2</td><td>1145.609466</td><td>1.004137931</td><td>5346.149921</td><td>725</td><td>2885</td></tr>
<tr><td>cancer care</td><td>cancer care</td><td>cancer care|&|Cancer Care|&|care cancer|&|care for cancer|&|cancer under the care|&|care on cancer</td><td>2</td><td>623.161193</td><td>1.306930693</td><td>1405.116897</td><td>303</td><td>2546</td></tr>
<tr><td>cancer cell</td><td>cancer cells</td><td>cancer cells|&|cancer cell|&|cell cancer|&|cells and cancer</td><td>2</td><td>1948.165548</td><td>1.354485777</td><td>10639.11253</td><td>1140</td><td>8986</td></tr>
<tr><td>cancer cell line</td><td>cancer cell lines</td><td>cancer cell lines</td><td>3</td><td>416.9994797</td><td>1.09375</td><td>30839.38302</td><td>192</td><td>1736</td></tr>
<tr><td>cancer center</td><td>Cancer Center</td><td>Cancer Center|&|cancer centers|&|cancer center|&|Center Cancer</td><td>2</td><td>445.3399435</td><td>1.150406504</td><td>1076.032896</td><td>308</td><td>3768</td></tr>
<tr><td>cancer colon</td><td>colon cancer</td><td>colon cancer|&|Colon Cancer|&|cancer of the colon|&|cancer in the colon</td><td>2</td><td>1014.997398</td><td>1.449438202</td><td>1605.908259</td><td>495</td><td>4838</td></tr>
<tr><td>cancer colorect metastat</td><td>metastatic colorectal cancer</td><td>metastatic colorectal cancer</td><td>3</td><td>790.3132995</td><td>1.363013699</td><td>9278.751389</td><td>292</td><td>3755</td></tr>
<tr><td>cancer death</td><td>cancer death</td><td>cancer death|&|cancer deaths|&|death from cancer|&|deaths from cancer|&|cancer and death|&|cancer or death</td><td>2</td><td>487.8282067</td><td>1.606217617</td><td>1129.812566</td><td>211</td><td>2715</td></tr>
<tr><td>cancer diagnosi</td><td>cancer diagnosis</td><td>cancer diagnosis|&|diagnosis of cancer|&|cancer at diagnosis|&|cancer with diagnosis|&|diagnosis with cancer|&|diagnosis for cancer|&|diagnosis of a cancer|&|diagnosis of any cancer</td><td>2</td><td>475.2390917</td><td>1.290598291</td><td>1256.385974</td><td>300</td><td>3687</td></tr>
<tr><td>cancer human</td><td>human cancers</td><td>human cancers|&|human cancer</td><td>2</td><td>698.6958831</td><td>1.126903553</td><td>5878.757016</td><td>394</td><td>2695</td></tr>
<tr><td>cancer incid</td><td>cancer incidence</td><td>cancer incidence|&|incidence of cancer|&|cancer and incidence|&|incidence of either cancer|&|incidence of any cancer</td><td>2</td><td>637.3239474</td><td>1.563706564</td><td>3050.539204</td><td>308</td><td>4034</td></tr>
<tr><td>cancer lung</td><td>lung cancer</td><td>lung cancer|&|Lung Cancer|&|lung cancers|&|Lung cancer|&|cancers of the lung|&|Cancer Lung|&|cancer of the lung</td><td>2</td><td>2609.094086</td><td>1.615984405</td><td>25070.71868</td><td>2696</td><td>29302</td></tr>
<tr><td>cancer lung non-small-cel</td><td>non-small-cell lung cancer</td><td>non-small-cell lung cancer|&|Non-Small-Cell Lung Cancer</td><td>3</td><td>2781.982243</td><td>1.097962382</td><td>24941.28262</td><td>1479</td><td>17842</td></tr>
<tr><td>cancer lung small-cel</td><td>small-cell lung cancer</td><td>small-cell lung cancer</td><td>3</td><td>548.056459</td><td>1.215859031</td><td>4433.334973</td><td>227</td><td>2742</td></tr>
<tr><td>cancer mortal</td><td>cancer mortality</td><td>cancer mortality|&|mortality from cancer|&|mortality of cancer|&|mortality in cancer|&|cancer and mortality|&|mortality from this cancer</td><td>2</td><td>448.4872223</td><td>1.484375</td><td>1379.350869</td><td>251</td><td>3169</td></tr>
<tr><td>cancer patient</td><td>cancer patients</td><td>cancer patients|&|patients with cancer|&|Cancer Patients|&|Patients With Cancer|&|cancer in patients|&|patients without cancer|&|Patients with Cancer|&|patients with this cancer|&|cancer in these patients|&|Cancer in Patients|&|Patients Cancer|&</td><td>2</td><td>2214.110603</td><td>1.292011019</td><td>9226.345837</td><td>1643</td><td>16649</td></tr>
<tr><td>cancer prostat</td><td>prostate cancer</td><td>prostate cancer|&|Prostate Cancer|&|prostate cancers|&|Prostate cancer|&|cancer of the prostate|&|cancers of the prostate</td><td>2</td><td>4286.593661</td><td>1.731722823</td><td>1314.21456</td><td>1875</td><td>17155</td></tr>
<tr><td>cancer registri</td><td>Cancer Registry</td><td>Cancer Registry|&|cancer registries|&|cancer registry</td><td>2</td><td>459.5026979</td><td>1.081481481</td><td>1143.11303</td><td>270</td><td>3369</td></tr>
<tr><td>cancer risk</td><td>cancer risk</td><td>cancer risk|&|Cancer Risk|&|risk of cancer|&|Risk of Cancer|&|risk for cancer|&|risk of any cancer|&|risk for any cancer|&|Cancer at Risk|&|risk of another cancer</td><td>2</td><td>780.5251307</td><td>1.30183727</td><td>2119.788082</td><td>612</td><td>6628</td></tr>
<tr><td>cancer survivor</td><td>cancer survivors</td><td>cancer survivors|&|Cancer Survivor|&|Cancer Survivors|&|survivors of cancer|&|Survivors of Cancer|&|survivors of any cancer|&|cancer and survivors</td><td>2</td><td>676.6649318</td><td>1.569343066</td><td>1546.867592</td><td>516</td><td>5196</td></tr>
<tr><td>cancer therapi</td><td>cancer therapy</td><td>cancer therapy|&|cancer therapies|&|therapy for cancer</td><td>2</td><td>525.5955517</td><td>1.098684211</td><td>1421.432456</td><td>304</td><td>2531</td></tr>
<tr><td>cancer treatment</td><td>cancer treatment</td><td>cancer treatment|&|Cancer Treatment|&|Treatment of Cancer|&|treatment of cancer|&|treatment for cancer|&|treatment of this cancer|&|Cancer and Treatment|&|cancer for the treatment|&|cancer after treatment</td><td>2</td><td>911.1371989</td><td>1.10707457</td><td>1199.500687</td><td>649</td><td>6737</td></tr>
<tr><td>candid gene</td><td>candidate genes</td><td>candidate genes|&|candidate gene|&|gene candidate|&|gene a candidate</td><td>2</td><td>623.161193</td><td>1.112359551</td><td>3099.122916</td><td>356</td><td>2212</td></tr>
<tr><td>carbon dioxid</td><td>carbon dioxide</td><td>carbon dioxide|&|dioxide carbon</td><td>2</td><td>884.3853295</td><td>1.28604119</td><td>7278.555328</td><td>545</td><td>1945</td></tr>
<tr><td>carbon nanotub</td><td>carbon nanotubes</td><td>carbon nanotubes|&|carbon nanotube|&|nanotube carbon</td><td>2</td><td>958.3463802</td><td>1.282105263</td><td>1316.968643</td><td>559</td><td>1852</td></tr>
<tr><td>carcinoma cell</td><td>cell carcinoma</td><td>cell carcinoma|&|Cell Carcinoma|&|cell carcinomas|&|carcinoma cell</td><td>2</td><td>907.9899202</td><td>1.140316206</td><td>1611.606401</td><td>1010</td><td>10667</td></tr>
<tr><td>carcinoma cell renal</td><td>renal cell carcinoma</td><td>renal cell carcinoma|&|cell renal carcinoma</td><td>3</td><td>440.8280214</td><td>1.150259067</td><td>12260.18753</td><td>277</td><td>3392</td></tr>
<tr><td>carcinoma cell squamou</td><td>squamous cell carcinoma</td><td>squamous cell carcinoma</td><td>3</td><td>599.684966</td><td>1.170542636</td><td>11200.57921</td><td>258</td><td>3313</td></tr>
<tr><td>cardiac death</td><td>cardiac death</td><td>cardiac death</td><td>2</td><td>396.5571228</td><td>1.37704918</td><td>2206.825423</td><td>184</td><td>2554</td></tr>
<tr><td>cardiolog european societi</td><td>European Society of Cardiology</td><td>European Society of Cardiology|&|Cardiology European Society</td><td>4</td><td>1828.840575</td><td>1.006557377</td><td>8786.08366</td><td>915</td><td>9246</td></tr>
<tr><td>cardiomyopathi dilat</td><td>dilated cardiomyopathy</td><td>dilated cardiomyopathy</td><td>2</td><td>497.2700429</td><td>1.284552846</td><td>1443.924818</td><td>246</td><td>1892</td></tr>
<tr><td>cardiovascular death</td><td>cardiovascular death</td><td>cardiovascular death</td><td>2</td><td>621.5875537</td><td>1.42599278</td><td>2721.413187</td><td>277</td><td>4315</td></tr>
<tr><td>cardiovascular diseas</td><td>cardiovascular disease</td><td>cardiovascular disease|&|cardiovascular diseases</td><td>2</td><td>2730.264318</td><td>1.33976834</td><td>5428.70112</td><td>1374</td><td>15775</td></tr>
<tr><td>cardiovascular event</td><td>cardiovascular events</td><td>cardiovascular events</td><td>2</td><td>1112.563039</td><td>1.38627451</td><td>2464.212538</td><td>544</td><td>7526</td></tr>
<tr><td>cardiovascular factor risk</td><td>cardiovascular risk factors</td><td>cardiovascular risk factors</td><td>3</td><td>563.9421534</td><td>1.198312236</td><td>3896.601957</td><td>237</td><td>3084</td></tr>
<tr><td>cardiovascular mortal</td><td>cardiovascular mortality</td><td>cardiovascular mortality</td><td>2</td><td>613.7193568</td><td>1.488549618</td><td>2065.640337</td><td>272</td><td>3560</td></tr>
<tr><td>cardiovascular risk</td><td>cardiovascular risk</td><td>cardiovascular risk|&|risk cardiovascular</td><td>2</td><td>468.9445342</td><td>1.150579151</td><td>1686.125311</td><td>259</td><td>3145</td></tr>
<tr><td>care health</td><td>health care</td><td>health care|&|Health Care|&|care health</td><td>2</td><td>2423.40464</td><td>1.37254902</td><td>1993.787456</td><td>1323</td><td>10951</td></tr>
<tr><td>care intens unit</td><td>intensive care unit</td><td>intensive care unit|&|intensive care units</td><td>3</td><td>911.4417198</td><td>1.217506631</td><td>26177.82897</td><td>377</td><td>4679</td></tr>
<tr><td>care primari</td><td>primary care</td><td>primary care</td><td>2</td><td>898.5480839</td><td>1.280269058</td><td>1398.220788</td><td>446</td><td>4118</td></tr>
<tr><td>care qualiti</td><td>quality of care</td><td>quality of care|&|care quality|&|quality care|&|quality for the care|&|quality of the care|&|care and quality</td><td>3</td><td>457.9290585</td><td>1.310810811</td><td>1394.60032</td><td>222</td><td>2090</td></tr>
<tr><td>care unit</td><td>care unit</td><td>care unit|&|care units|&|unit care</td><td>2</td><td>468.9445342</td><td>1.216326531</td><td>2530.280631</td><td>589</td><td>7032</td></tr>
<tr><td>care usual</td><td>usual care</td><td>usual care</td><td>2</td><td>1057.485661</td><td>1.92</td><td>2842.566401</td><td>350</td><td>4624</td></tr>
<tr><td>carrier mutat</td><td>mutation carriers</td><td>mutation carriers|&|carriers of a mutation|&|carriers of this mutation|&|carriers of the mutation</td><td>2</td><td>582.2465693</td><td>1.787439614</td><td>1394.50052</td><td>237</td><td>2305</td></tr>
<tr><td>case-control studi</td><td>case-control study</td><td>case-control study|&|case-control studies</td><td>2</td><td>1104.694842</td><td>1.114285714</td><td>3238.744244</td><td>727</td><td>6923</td></tr>
<tr><td>caus death</td><td>death from any cause</td><td>death from any cause|&|cause of death|&|causes of death|&|death from all causes|&|cause death|&|causes death|&|death from causes|&|death of any cause|&|cause and death|&|death causes|&|death for any cause|&|cause the death|&|death for a</td><td>4</td><td>1060.63294</td><td>1.281368821</td><td>1401.607677</td><td>535</td><td>7303</td></tr>
<tr><td>caus mortal</td><td>cause mortality</td><td>cause mortality|&|mortality from any cause|&|cause of mortality|&|mortality by cause|&|mortality cause</td><td>2</td><td>465.7972554</td><td>1.85</td><td>1640.437697</td><td>160</td><td>2409</td></tr>
<tr><td>cd4 cell count</td><td>CD4 cell count</td><td>CD4 cell count|&|CD4 cell counts</td><td>3</td><td>476.5708339</td><td>1.983471074</td><td>1261.729137</td><td>121</td><td>1661</td></tr>
<tr><td>cell cultur</td><td>cell culture</td><td>cell culture|&|cultured cells</td><td>2</td><td>568.0838149</td><td>1.046376812</td><td>1907.605199</td><td>345</td><td>1746</td></tr>
<tr><td>cell cycl</td><td>cell cycle</td><td>cell cycle</td><td>2</td><td>1306.120682</td><td>1.263318113</td><td>9222.787491</td><td>806</td><td>4873</td></tr>
<tr><td>cell death</td><td>cell death</td><td>cell death|&|death cell</td><td>2</td><td>1528.003834</td><td>1.254521964</td><td>4925.218713</td><td>841</td><td>4881</td></tr>
<tr><td>cell dendrit</td><td>dendritic cells</td><td>dendritic cells|&|dendritic cell|&|cell dendritic</td><td>2</td><td>1139.314909</td><td>1.288256228</td><td>8628.618588</td><td>562</td><td>3385</td></tr>
<tr><td>cell differenti</td><td>cell differentiation</td><td>cell differentiation|&|differentiated cells</td><td>2</td><td>618.4402749</td><td>1.11965812</td><td>4557.048939</td><td>351</td><td>1971</td></tr>
<tr><td>cell divis</td><td>cell division</td><td>cell division|&|cell divisions|&|divisions that each cell|&|division per cell</td><td>2</td><td>1046.470185</td><td>1.238361266</td><td>4577.267766</td><td>537</td><td>2382</td></tr>
<tr><td>cell endotheli</td><td>endothelial cells</td><td>endothelial cells</td><td>2</td><td>863.9280176</td><td>1.369077307</td><td>2245.522275</td><td>401</td><td>2402</td></tr>
<tr><td>cell epitheli</td><td>epithelial cells</td><td>epithelial cells</td><td>2</td><td>610.572078</td><td>1.208722741</td><td>1581.805193</td><td>321</td><td>1767</td></tr>
<tr><td>cell es</td><td>ES cells</td><td>ES cells|&|ES cell</td><td>2</td><td>676.6649318</td><td>2.216494845</td><td>3980.53482</td><td>194</td><td>939</td></tr>
<tr><td>cell fate</td><td>cell fate</td><td>cell fate|&|cell fates|&|fate of a cell|&|fate of cell</td><td>2</td><td>810.4242788</td><td>1.2004662</td><td>6588.861803</td><td>429</td><td>2149</td></tr>
<tr><td>cell germ</td><td>germ cell</td><td>germ cell|&|germ cells|&|Germ Cell</td><td>2</td><td>919.0053958</td><td>1.420924574</td><td>5730.601973</td><td>514</td><td>3746</td></tr>
<tr><td>cell growth</td><td>cell growth</td><td>cell growth</td><td>2</td><td>626.3084718</td><td>1.07277628</td><td>4417.032389</td><td>371</td><td>2291</td></tr>
<tr><td>cell host</td><td>host cells</td><td>host cells|&|host cell</td><td>2</td><td>632.6030293</td><td>1.252336449</td><td>2598.676428</td><td>321</td><td>1320</td></tr>
<tr><td>cell human</td><td>human cells</td><td>human cells</td><td>2</td><td>915.8581171</td><td>1.100189036</td><td>3742.168175</td><td>529</td><td>2484</td></tr>
<tr><td>cell line</td><td>cell lines</td><td>cell lines|&|cell line</td><td>2</td><td>1833.289873</td><td>1.181541582</td><td>11452.509</td><td>1136</td><td>7471</td></tr>
<tr><td>cell live</td><td>living cells</td><td>living cells</td><td>2</td><td>621.5875537</td><td>1.131805158</td><td>2290.509475</td><td>349</td><td>1435</td></tr>
<tr><td>cell mammalian</td><td>mammalian cells</td><td>mammalian cells</td><td>2</td><td>1010.27648</td><td>1.07</td><td>5977.230124</td><td>600</td><td>2687</td></tr>
<tr><td>cell membran</td><td>cell membrane</td><td>cell membrane|&|cell membranes|&|membranes of the cell|&|membrane at the cell</td><td>2</td><td>396.5571228</td><td>1.125</td><td>3335.703691</td><td>224</td><td>1028</td></tr>
</table>

<table>
<tr><th></th><th></th><th></th><th></th><th></th><th></th><th></th><th></th><th></th></tr>
<tr><td>cell popul</td><td>cell populations</td><td>cell populations|&|cell population</td><td>2</td><td>725.4477525</td><td>1.127139364</td><td>4574.66855</td><td>409</td><td>2094</td></tr>
<tr><td>cell progenitor</td><td>progenitor cells</td><td>progenitor cells|&|progenitor cell|&|cell progenitor</td><td>2</td><td>1428.864554</td><td>1.32748538</td><td>3773.0413</td><td>684</td><td>4052</td></tr>
<tr><td>cell prolifer</td><td>cell proliferation</td><td>cell proliferation</td><td>2</td><td>963.0672983</td><td>1.112727273</td><td>5229.20862</td><td>550</td><td>3808</td></tr>
<tr><td>cell singl</td><td>single cells</td><td>single cells</td><td>2</td><td>440.6190254</td><td>1.142857143</td><td>2302.342547</td><td>245</td><td>1115</td></tr>
<tr><td>cell surfac</td><td>cell surface</td><td>cell surface|&|surface cell</td><td>2</td><td>760.0678188</td><td>1.139150943</td><td>2485.199596</td><td>424</td><td>2031</td></tr>
<tr><td>cell t</td><td>T cells</td><td>T cells|&|T cell|&|T Cells|&|cells T</td><td>2</td><td>5554.946999</td><td>1.765882941</td><td>13967.76355</td><td>2348</td><td>14437</td></tr>
<tr><td>cell tumor</td><td>tumor cells</td><td>tumor cells|&|tumor cell|&|cell tumors|&|cell tumor|&|cells within a tumor|&|cells in the tumor</td><td>2</td><td>1814.406201</td><td>1.351699883</td><td>2981.447793</td><td>966</td><td>8900</td></tr>
<tr><td>cell tumour</td><td>tumour cells</td><td>tumour cells|&|tumour cell|&|cell tumour|&|cells within a tumour</td><td>2</td><td>706.56408</td><td>1.522033898</td><td>2528.217005</td><td>295</td><td>2384</td></tr>
<tr><td>cell type</td><td>cell types</td><td>cell types|&|cell type|&|type of cell|&|type cell</td><td>2</td><td>1899.382727</td><td>1.231632653</td><td>6569.347028</td><td>1030</td><td>4941</td></tr>
<tr><td>central nervou system</td><td>central nervous system</td><td>central nervous system</td><td>3</td><td>990.8701921</td><td>1.063965885</td><td>1292.192088</td><td>469</td><td>2336</td></tr>
<tr><td>central role</td><td>central role</td><td>central role</td><td>2</td><td>535.037388</td><td>1.00591716</td><td>1709.789356</td><td>338</td><td>1650</td></tr>
<tr><td>cerevisia saccharomyc</td><td>Saccharomyces cerevisiae</td><td>Saccharomyces cerevisiae</td><td>2</td><td>859.2070995</td><td>1.001834862</td><td>5029.901892</td><td>679</td><td>2937</td></tr>
<tr><td>chain polymeras reaction</td><td>polymerase chain reaction</td><td>polymerase chain reaction</td><td>3</td><td>867.7560601</td><td>1.030660377</td><td>1600.906704</td><td>550</td><td>5983</td></tr>
<tr><td>chang climat</td><td>climate change</td><td>climate change|&|climate changes|&|change in climate|&|changes in climate|&|change with climate|&|climate of change|&|changes in the climate|&|change from a climate</td><td>2</td><td>1254.190583</td><td>1.261075949</td><td>7465.934189</td><td>632</td><td>2140</td></tr>
<tr><td>chang conform</td><td>conformational changes</td><td>conformational changes|&|conformational change|&|changes conformational</td><td>2</td><td>670.3703743</td><td>1.097938144</td><td>10288.41582</td><td>388</td><td>1549</td></tr>
<tr><td>channel ion</td><td>ion channels</td><td>ion channels|&|ion channel|&|channel ion</td><td>2</td><td>862.3543783</td><td>1.330097087</td><td>2397.715935</td><td>412</td><td>1659</td></tr>
<tr><td>chemotherapi high-dos</td><td>high-dose chemotherapy</td><td>high-dose chemotherapy</td><td>2</td><td>402.8516804</td><td>1.267326733</td><td>2267.716591</td><td>202</td><td>2275</td></tr>
<tr><td>chemotherapi induct</td><td>induction chemotherapy</td><td>induction chemotherapy|&|chemotherapy induction|&|chemotherapy during induction</td><td>2</td><td>525.5955517</td><td>1.546296296</td><td>1474.529962</td><td>216</td><td>2730</td></tr>
<tr><td>chemotherapi neoadjuv</td><td>neoadjuvant chemotherapy</td><td>neoadjuvant chemotherapy|&|chemotherapy in the neoadjuvant</td><td>2</td><td>544.4792242</td><td>1.655502392</td><td>1088.554416</td><td>209</td><td>2525</td></tr>
<tr><td>chemotherapi regimen</td><td>chemotherapy regimens</td><td>chemotherapy regimens|&|chemotherapy regimen|&|regimen of chemotherapy|&|regimens of chemotherapy|&|regimen for chemotherapy|&|regimen with chemotherapy</td><td>2</td><td>782.0987701</td><td>1.129545455</td><td>2377.81637</td><td>440</td><td>6489</td></tr>
<tr><td>chest pain</td><td>chest pain</td><td>chest pain|&|pain in the chest</td><td>2</td><td>478.3863704</td><td>1.490196078</td><td>1163.510839</td><td>204</td><td>2252</td></tr>
<tr><td>cholesterol ldl</td><td>LDL cholesterol</td><td>LDL cholesterol</td><td>2</td><td>506.7118792</td><td>1.916666667</td><td>1211.455039</td><td>182</td><td>2332</td></tr>
<tr><td>cholesterol level</td><td>cholesterol levels</td><td>cholesterol levels|&|cholesterol level|&|levels of cholesterol</td><td>2</td><td>580.6729299</td><td>1.563559322</td><td>1948.087501</td><td>251</td><td>3577</td></tr>
<tr><td>chromosom segreg</td><td>chromosome segregation</td><td>chromosome segregation</td><td>2</td><td>437.4717466</td><td>1.252252252</td><td>4497.72119</td><td>222</td><td>906</td></tr>
<tr><td>chromosom x</td><td>X chromosome</td><td>X chromosome|&|X chromosomes|&|chromosome X|&|chromosomes X</td><td>2</td><td>640.4712262</td><td>1.674897119</td><td>1224.285674</td><td>243</td><td>954</td></tr>
<tr><td>chronic failur heart</td><td>chronic heart failure</td><td>chronic heart failure</td><td>3</td><td>534.1564763</td><td>1.120833333</td><td>6837.84787</td><td>340</td><td>3402</td></tr>
<tr><td>circadian clock</td><td>circadian clock</td><td>circadian clock</td><td>2</td><td>420.1617135</td><td>1.296116505</td><td>1273.507379</td><td>206</td><td>958</td></tr>
<tr><td>class ii</td><td>class II</td><td>class II|&|class of II</td><td>2</td><td>451.634501</td><td>1.292792793</td><td>1375.522277</td><td>222</td><td>1909</td></tr>
<tr><td>clin j oncol</td><td>J Clin Oncol</td><td>J Clin Oncol</td><td>3</td><td>2942.824899</td><td>1</td><td>15619.54314</td><td>1482</td><td>20631</td></tr>
<tr><td>cohort prospect studi</td><td>prospective cohort study</td><td>prospective cohort study|&|Prospective cohort study|&|prospective cohort studies|&|study of a prospective cohort|&|prospective study in a cohort</td><td>3</td><td>1247.027015</td><td>1.021138211</td><td>11007.09834</td><td>719</td><td>8685</td></tr>
<tr><td>cohort retrospect studi</td><td>retrospective cohort study</td><td>retrospective cohort study|&|Retrospective cohort study</td><td>3</td><td>955.1273796</td><td>1.006276151</td><td>7680.245704</td><td>478</td><td>5829</td></tr>
<tr><td>cohort studi</td><td>cohort study</td><td>cohort study|&|cohort studies|&|study cohort|&|Cohort Study|&|Cohort study|&|Study Cohort|&|study of a cohort|&|study a cohort</td><td>2</td><td>2736.558876</td><td>1.055218447</td><td>15880.43581</td><td>3193</td><td>35442</td></tr>
<tr><td>coli escherichia</td><td>Escherichia coli</td><td>Escherichia coli</td><td>2</td><td>1753.034265</td><td>1.007233273</td><td>5987.686391</td><td>1106</td><td>4537</td></tr>
<tr><td>colony-stimul factor granulocyt</td><td>granulocyte colony-stimulating factor</td><td>granulocyte colony-stimulating factor|&|colony-stimulating factor and granulocyte</td><td>3</td><td>393.170938</td><td>1.010204082</td><td>1394.25088</td><td>196</td><td>2265</td></tr>
<tr><td>complet remiss</td><td>complete remission</td><td>complete remission</td><td>2</td><td>698.6958831</td><td>1.206521739</td><td>3889.401143</td><td>368</td><td>4713</td></tr>
<tr><td>complet respons</td><td>complete response</td><td>complete response|&|complete responses</td><td>2</td><td>1403.686324</td><td>1.145057766</td><td>12497.05429</td><td>789</td><td>11622</td></tr>
<tr><td>complex protein</td><td>protein complexes</td><td>protein complexes</td><td>2</td><td>420.1617135</td><td>1.197309417</td><td>4675.134872</td><td>223</td><td>1030</td></tr>
<tr><td>comput tomographi</td><td>computed tomography</td><td>computed tomography</td><td>2</td><td>708.1377194</td><td>1.061320755</td><td>1680.182246</td><td>481</td><td>4363</td></tr>
<tr><td>conclus patient</td><td>Conclusion Patients</td><td>Conclusion Patients|&|Conclusion In patients|&|CONCLUSIONS In patients|&|Conclusion Among patients|&|CONCLUSIONS Among patients|&|Conclusions In patients|&|Conclusions Among patients|&|Conclusion For patients|&|Conclusions For p</td><td>2</td><td>1419.422717</td><td>1</td><td>1628.870365</td><td>902</td><td>12619</td></tr>
<tr><td>conclus relev</td><td>CONCLUSIONS AND RELEVANCE</td><td>CONCLUSIONS AND RELEVANCE</td><td>3</td><td>1302.973404</td><td>1</td><td>10114.83371</td><td>828</td><td>11905</td></tr>
<tr><td>confid interv</td><td>confidence intervals</td><td>confidence intervals|&|confidence interval</td><td>2</td><td>804.1297213</td><td>1.071278826</td><td>3313.937433</td><td>477</td><td>5819</td></tr>
<tr><td>confid percent</td><td>percent confidence</td><td>percent confidence</td><td>2</td><td>1680.646854</td><td>1.561403509</td><td>16650.29352</td><td>684</td><td>7707</td></tr>
<tr><td>congenit diseas heart</td><td>congenital heart disease</td><td>congenital heart disease</td><td>3</td><td>460.6851394</td><td>1.171717172</td><td>7181.627859</td><td>198</td><td>1563</td></tr>
<tr><td>congest failur heart</td><td>congestive heart failure</td><td>congestive heart failure</td><td>3</td><td>941.2273969</td><td>1.281081081</td><td>5339.414876</td><td>370</td><td>4815</td></tr>
<tr><td>consecut patient</td><td>consecutive patients</td><td>consecutive patients</td><td>2</td><td>1166.066778</td><td>1.027739251</td><td>3112.709927</td><td>721</td><td>7942</td></tr>
<tr><td>control group</td><td>control group</td><td>control group|&|control groups|&|group against control</td><td>2</td><td>4601.321537</td><td>2.11884058</td><td>2513.905218</td><td>1380</td><td>16234</td></tr>
<tr><td>control randomis trial</td><td>randomised controlled trials</td><td>randomised controlled trials|&|Randomised controlled trials|&|controlled randomised trials</td><td>3</td><td>969.0273623</td><td>1.308310992</td><td>6978.438139</td><td>373</td><td>4009</td></tr>
<tr><td>cooper eastern group oncolog</td><td>Eastern Cooperative Oncology Group</td><td>Eastern Cooperative Oncology Group</td><td>4</td><td>891.5846017</td><td>1.004474273</td><td>14279.51565</td><td>447</td><td>8606</td></tr>
<tr><td>cooper group oncolog perform</td><td>Cooperative Oncology Group performance</td><td>Cooperative Oncology Group performance</td><td>4</td><td>587.7706951</td><td>1.003389831</td><td>10357.37269</td><td>295</td><td>6269</td></tr>
<tr><td>copi number</td><td>copy number</td><td>copy number</td><td>2</td><td>988.2455284</td><td>1.322105263</td><td>1483.519382</td><td>525</td><td>3729</td></tr>
<tr><td>coronari diseas heart</td><td>coronary heart disease</td><td>coronary heart disease</td><td>3</td><td>2233.925784</td><td>1.478318003</td><td>20911.18408</td><td>823</td><td>9784</td></tr>
<tr><td>coronari intervent percutan</td><td>percutaneous coronary intervention</td><td>percutaneous coronary intervention</td><td>3</td><td>1358.226877</td><td>1.11038961</td><td>35011.19204</td><td>794</td><td>9847</td></tr>
<tr><td>coronari intervent percutan primari</td><td>primary percutaneous coronary intervention</td><td>primary percutaneous coronary intervention|&|coronary intervention vs. primary percutaneous intervention</td><td>4</td><td>391.1852262</td><td>1.059139785</td><td>15467.74186</td><td>186</td><td>2545</td></tr>
<tr><td>cox hazard model proport</td><td>Cox proportional hazards models</td><td>Cox proportional hazards models|&|Cox proportional hazards model</td><td>4</td><td>712.870539</td><td>1.011267606</td><td>10401.41412</td><td>355</td><td>5842</td></tr>
<tr><td>cox hazard proport</td><td>Cox proportional hazards</td><td>Cox proportional hazards|&|Cox proportional hazard</td><td>3</td><td>480.5422575</td><td>1.016806723</td><td>2203.242331</td><td>782</td><td>12133</td></tr>
<tr><td>cox hazard proport regress</td><td>Cox proportional hazards regression</td><td>Cox proportional hazards regression|&|Cox regression proportional hazards</td><td>4</td><td>405.0852088</td><td>1.02</td><td>7416.813098</td><td>200</td><td>3082</td></tr>
<tr><td>cox multivari</td><td>multivariate Cox</td><td>multivariate Cox|&|multivariable Cox|&|Cox multivariate|&|Cox multivariable</td><td>2</td><td>456.3554192</td><td>1.028368794</td><td>3056.632529</td><td>282</td><td>4386</td></tr>
<tr><td>cox regress</td><td>Cox regression</td><td>Cox regression|&|regression Cox</td><td>2</td><td>467.3708948</td><td>1.03125</td><td>5117.625372</td><td>635</td><td>9498</td></tr>
<tr><td>c-reactiv protein</td><td>C-reactive protein</td><td>C-reactive protein</td><td>2</td><td>974.082774</td><td>1.628947368</td><td>1986.593462</td><td>410</td><td>5006</td></tr>
<tr><td>criteria elig</td><td>eligibility criteria</td><td>eligibility criteria|&|Eligibility criteria</td><td>2</td><td>420.1617135</td><td>1.145922747</td><td>1236.190636</td><td>233</td><td>3253</td></tr>
<tr><td>criteria inclus</td><td>inclusion criteria</td><td>inclusion criteria|&|criteria for inclusion</td><td>2</td><td>505.1382398</td><td>1.049019608</td><td>2178.275158</td><td>306</td><td>3738</td></tr>
<tr><td>critic role</td><td>critical role</td><td>critical role|&|critical roles</td><td>2</td><td>884.3853295</td><td>1.016274864</td><td>2431.372164</td><td>553</td><td>2588</td></tr>
<tr><td>crucial role</td><td>crucial role</td><td>crucial role</td><td>2</td><td>481.5336492</td><td>1.00990099</td><td>1390.197205</td><td>303</td><td>1406</td></tr>
<tr><td>cryo-electron microscopi</td><td>cryo-electron microscopy</td><td>cryo-electron microscopy</td><td>2</td><td>489.4018461</td><td>1.023026316</td><td>12795.63145</td><td>304</td><td>1082</td></tr>
<tr><td>crystal report structur</td><td>report the crystal structure</td><td>report the crystal structure|&|report crystal structures|&|report the crystal structures|&|report a crystal structure|&|report on the crystal structure</td><td>4</td><td>603.6563896</td><td>1</td><td>64240.96875</td><td>304</td><td>1488</td></tr>
<tr><td>crystal structur</td><td>crystal structure</td><td>crystal structure|&|crystal structures|&|Crystal structure|&|Crystal structures</td><td>2</td><td>2264.467063</td><td>1.054212454</td><td>41960.44058</td><td>1720</td><td>7114</td></tr>
<tr><td>damag dna</td><td>DNA damage</td><td>DNA damage|&|damage DNA|&|damage the DNA|&|DNA from damage</td><td>2</td><td>1406.833602</td><td>1.425837321</td><td>6696.798871</td><td>739</td><td>4306</td></tr>
<tr><td>data set</td><td>data sets</td><td>data sets|&|sets of data</td><td>2</td><td>837.1761482</td><td>1.156521739</td><td>1309.766103</td><td>460</td><td>2575</td></tr>
<tr><td>data sourc</td><td>Data sources</td><td>Data sources|&|Data Sources|&|data sources|&|DATA SOURCES|&|sources Data</td><td>2</td><td>782.0987701</td><td>1.026859504</td><td>5458.809802</td><td>562</td><td>6146</td></tr>
<tr><td>death hazard ratio</td><td>hazard ratio for death</td><td>hazard ratio for death|&|hazard ratio of death|&|death hazard ratio</td><td>4</td><td>403.099497</td><td>1.153409091</td><td>1989.750271</td><td>176</td><td>2618</td></tr>
<tr><td>death rate</td><td>death rates</td><td>death rates|&|death rate|&|rate of death|&|rates of death</td><td>2</td><td>1104.694842</td><td>1.5</td><td>2050.083997</td><td>468</td><td>5784</td></tr>
<tr><td>death risk</td><td>risk of death</td><td>risk of death|&|death risk|&|risk for death|&|death and risk|&|risk until death</td><td>3</td><td>843.4707057</td><td>1.240740741</td><td>1391.395471</td><td>437</td><td>6204</td></tr>
<tr><td>depart emerg</td><td>emergency department</td><td>emergency department|&|emergency departments|&|department emergency</td><td>2</td><td>889.1062477</td><td>1.482939633</td><td>1922.881868</td><td>408</td><td>4058</td></tr>
<tr><td>diabet mellitu</td><td>diabetes mellitus</td><td>diabetes mellitus</td><td>2</td><td>1332.872552</td><td>1.206552707</td><td>1214.329613</td><td>702</td><td>7414</td></tr>
<tr><td>differ risk</td><td>risk difference</td><td>risk difference|&|difference in risk|&|difference in the risk</td><td>2</td><td>423.3089922</td><td>1.393782383</td><td>1608.097714</td><td>259</td><td>3546</td></tr>
<tr><td>differ signific</td><td>significant differences</td><td>significant differences|&|significant difference</td><td>2</td><td>3668.153386</td><td>1.134858812</td><td>3421.212622</td><td>2121</td><td>26824</td></tr>
<tr><td>discharg hospit</td><td>hospital discharge</td><td>hospital discharge</td><td>2</td><td>530.3164698</td><td>1.484581498</td><td>1319.997842</td><td>227</td><td>2968</td></tr>
<tr><td>diseas graft-versus-host</td><td>graft-versus-host disease</td><td>graft-versus-host disease</td><td>2</td><td>426.456271</td><td>1.138655462</td><td>3809.460551</td><td>238</td><td>2467</td></tr>
<tr><td>diseas heart</td><td>heart disease</td><td>heart disease</td><td>2</td><td>1110.9894</td><td>1.329566855</td><td>22019.63439</td><td>1861</td><td>20013</td></tr>
<tr><td>diseas heart ischaem</td><td>ischaemic heart disease</td><td>ischaemic heart disease</td><td>3</td><td>732.7276571</td><td>1.470119522</td><td>6608.736076</td><td>251</td><td>2928</td></tr>
<tr><td>diseas human</td><td>human disease</td><td>human disease|&|human diseases</td><td>2</td><td>629.4557506</td><td>1.049868766</td><td>1744.713547</td><td>381</td><td>1860</td></tr>
<tr><td>diseas metastat</td><td>metastatic disease</td><td>metastatic disease|&|disease as metastatic</td><td>2</td><td>797.8351638</td><td>1.201421801</td><td>1443.734583</td><td>422</td><td>5496</td></tr>
</table>

diseas neurodegen	neurodegenerative diseases	neurodegenerative diseases|&|neurodegenerative disease	2	476.812731	1.165384615	1207.358435	260	1280
diseas progress	disease progression	disease progression|&|progressive disease|&|progression of disease|&|progression of the disease|&|disease progressive|&|progression disease	2	3054.43403	1.258754864	6576.354224	1585	22635
diseas risk	disease risk	disease risk|&|risk of disease|&|risk disease|&|risk of the disease|&|risk for the disease|&|risk for disease|&|disease and risk|&|disease with risk|&|risk of this disease	2	456.3554192	1.169354839	1663.229117	291	3065
diseas stabl	stable disease	stable disease	2	980.3773315	1.1125	11554.94925	560	8947
dna methyl	DNA methylation	DNA methylation|&|methylation of DNA|&|DNA from methylation	2	1381.655372	1.65037594	2936.613535	532	2800
dna polymeras	DNA polymerase	DNA polymerase|&|DNA polymerases	2	407.5725985	1.282178218	2342.094202	202	909
dna repair	DNA repair	DNA repair|&|repair DNA|&|repair of DNA|&|DNA during repair|&|repair the DNA	2	953.6254621	1.267782427	10687.31682	478	2865
dna replic	DNA replication	DNA replication|&|replication of DNA|&|replication before DNA|&|DNA during replication	2	944.1836258	1.34529148	8379.313253	446	2353
dna sequenc	DNA sequence	DNA sequence|&|DNA sequences|&|sequence DNA|&|sequence the DNA	2	841.8970664	1.126315789	2941.560748	475	2056
dose escal	dose escalation	dose escalation|&|escalating doses|&|escalation of the dose	2	426.456271	1.110655738	1908.471859	244	2941
dose level	dose levels	dose levels|&|dose level	2	714.4322769	1.455128205	3349.080248	312	3988
dose maximum-toler	maximum-tolerated dose	maximum-tolerated dose	2	516.1537155	1.071895425	5066.444229	306	3798
dose-limit toxic	dose-limiting toxicity	dose-limiting toxicity|&|dose-limiting toxicities	2	546.0528636	1.101587302	4188.294911	315	4120
dot quantum	quantum dots	quantum dots|&|quantum dot	2	1024.439234	1.557416268	4504.272039	418	1457
drosophila melanogast	Drosophila melanogaster	Drosophila melanogaster	2	805.7033607	1.001956947	4460.66666	511	1949
drug resist	drug resistance	drug resistance	2	643.6185049	1.31511254	1100.726432	311	2213
drug studi	study drug	study drug|&|drug under study|&|study of a drug	2	550.7737817	1.151315789	3920.851473	430	6842
drug-elut stent	drug-eluting stents	drug-eluting stents|&|drug-eluting stent	2	481.5336492	1.291139241	2834.680091	237	2527
durat median	median duration	median duration|&|Median duration	2	525.5955517	1.077419355	3082.291837	468	7109
efficaci safeti	efficacy and safety	efficacy and safety|&|safety and efficacy|&|Efficacy and safety|&|Safety and efficacy|&|efficacy or safety|&|safety or efficacy|&|efficacy and Safety	3	2643.714152	1.031941032	4606.139288	1628	21107
egfr mutat	EGFR mutations	EGFR mutations|&|EGFR mutation|&|mutations in EGFR|&|mutation in EGFR|&|mutations in the EGFR	2	446.9135829	2.308943089	4458.745651	123	2000
eject fraction	ejection fraction	ejection fraction	2	736.4632281	1.251336898	5272.724249	915	11681
eject fraction ventricular	ventricular ejection fraction	ventricular ejection fraction	3	951.155956	1.148681055	15146.70824	417	6196
electr field	electric field	electric field|&|electric fields	2	445.3399435	1.246696035	4939.441423	227	631
electron microscopi	electron microscopy	electron microscopy	2	667.2230956	1.087179487	3259.144004	536	1747
elev infarct myocardi st-segment	ST-segment elevation myocardial infarction	ST-segment elevation myocardial infarction|&|myocardial infarction with ST-segment elevation|&|myocardial infarction without ST-segment elevation	4	470.6136985	1.156097561	6155.416725	205	2981
elig patient	eligible patients	eligible patients|&|Eligible patients	2	1468.205538	1.082366589	3143.326291	971	15058
emiss positron tomographi	positron emission tomography	positron emission tomography	3	788.3275877	1.061497326	1123.175664	374	3332
encod gene	gene encoding	gene encoding|&|genes encoding|&|gene encodes|&|gene that encodes|&|encoding genes|&|encoding gene|&|encodes a gene	2	1194.392287	1.042582418	1836.946471	728	3963
end point	end point	end point|&|end points	2	1965.475581	1.286302781	24489.90258	3028	44632
end point primari	primary end point	primary end point|&|primary end points|&|Primary end point|&|Primary end points	3	4801.451152	1.142722117	24087.76662	2118	32233
end point secondari	Secondary end points	Secondary end points|&|secondary end points|&|secondary end point	3	1759.340662	1.041128085	12328.78777	851	14079
endoplasm reticulum	endoplasmic reticulum	endoplasmic reticulum	2	810.4242788	1.122004357	7046.322659	459	1923
endotheli factor growth vascular	vascular endothelial growth factor	vascular endothelial growth factor|&|factor vascular endothelial growth	4	496.427952	1.012145749	5259.994385	247	2712
endpoint primari	primary endpoint	primary endpoint|&|primary endpoints|&|Primary endpoints	2	3375.456462	1.175342466	8351.17846	1825	24703
endpoint secondari	Secondary endpoints	Secondary endpoints|&|secondary endpoints|&|secondary endpoint	2	508.2855186	1.059016393	3501.84548	305	4120
engl j med n	N Engl J Med	N Engl J Med	4	1681.897901	1	51174.23981	847	8491
enrol patient	enrolled patients	enrolled patients|&|patients enrolled	2	618.4402749	1.03968254	1896.688621	378	5582
epiderm factor growth receptor	epidermal growth factor receptor	epidermal growth factor receptor	4	691.0277091	1.008695652	17456.18825	345	4672
essenti role	essential role	essential role|&|essential roles	2	583.8202086	1.019230769	2712.37018	364	1785
estrogen receptor	estrogen receptor	estrogen receptor	2	572.804733	1.089820359	2891.450957	334	4242
event rate	event rates	event rates|&|event rate	2	613.7193568	1.368421053	1118.303491	285	4010
express gene	gene expression	gene expression|&|Gene expression|&|expressed genes|&|Gene Expression|&|expression of genes|&|expression of these genes|&|expression of the gene|&|expression of the genes|&|expression of this gene|&|genes for expression|&|genes expres	2	6472.378755	1.265927978	17422.73268	3471	19829
express level	expression levels	expression levels|&|expression level|&|level of expression|&|levels of expression|&|level expression	2	758.4941794	1.265091864	3011.487359	381	3243
express pattern	expression patterns	expression patterns|&|expression pattern|&|pattern of expression|&|patterns of expression	2	483.1072885	1.108303249	5084.327905	346	2123
express protein	protein expression	protein expression|&|expression of this protein	2	766.3623763	1.22979798	2021.461114	396	3111
factor growth	growth factor	growth factor|&|growth factors|&|Growth Factor	2	1661.763181	1.121019108	23796.98525	2522	25768
factor growth receptor	growth factor receptor	growth factor receptor|&|Growth Factor Receptor	3	1685.869325	1.027845036	23517.40399	1158	15185
factor prognost	prognostic factors	prognostic factors|&|prognostic factor|&|Prognostic factors|&|factors prognostic|&|factor prognostic	2	1926.134597	1.280334728	5130.96482	1081	13997
factor risk	risk factors	risk factors|&|risk factor|&|Risk factors|&|Risk Factors|&|risk by a factor|&|factors on the risk|&|risk and factors|&|factors with the risk	2	7668.344681	1.467771084	6510.038164	3662	39564
factor transcript	transcription factor	transcription factor|&|transcription factors|&|factor transcription|&|transcription by factors	2	4950.669478	1.213734568	20914.82995	2634	13572
failur heart	heart failure	heart failure|&|Heart Failure|&|Heart failure|&|heart from failure	2	5381.846667	1.52	6282.41706	3104	31551
failur heart patient	patients with heart failure	patients with heart failure|&|heart failure patients|&|heart failure in patients|&|patients without heart failure|&|heart failure in these patients|&|heart failure among patients|&|heart failure versus patients|&|heart failure for patients|&|heart failure in thos	4	818.1132649	1.263803681	6806.565895	341	4189
failur treatment	treatment failure	treatment failure|&|failure of treatment|&|failure treatment|&|failure in treatment|&|failure during treatment	2	955.1991015	1.421545667	1183.64151	427	5448
febril neutropenia	febrile neutropenia	febrile neutropenia	2	564.9365361	1.184818482	2159.204642	303	4753
feedback loop	feedback loop	feedback loop|&|feedback loops	2	468.9445342	1.226337449	1979.465535	243	1085
field magnet	magnetic field	magnetic field|&|magnetic fields	2	1164.493139	1.293706294	1631.61232	572	1272
first-lin therapi	first-line therapy	first-line therapy	2	409.1462379	1.140350877	1222.688431	228	3267
first-lin treatment	first-line treatment	first-line treatment	2	623.161193	1.125	2065.097743	417	5882
follow-up median	median follow-up	median follow-up|&|Median follow-up	2	3284.185379	1.048743719	3549.633124	2195	31619
follow-up median result	Results Median follow-up	Results Median follow-up|&|Results After a median follow-up|&|Results With a median follow-up|&|Results At a median follow-up|&|Results During a median follow-up|&|Results The median follow-up|&|Results Over a median follow-up|&|Results At	3	454.728004	1	2864.477691	229	3484
function ventricular	ventricular function	ventricular function	2	415.4407954	1.178571429	1792.992657	224	2512
fusion protein	fusion protein	fusion protein|&|fusion proteins|&|protein fusion	2	478.3863704	1.151515152	1255.429876	264	1564
g mu	mu g	mu g	2	660.9285381	1.52173913	1083.665239	276	2850
g protein	G protein	G protein|&|G proteins|&|protein G|&|proteins G	2	616.8666355	1.410071942	2315.353109	278	1129
gain weight	weight gain	weight gain|&|gain weight|&|gain in weight	2	549.2001424	1.608294931	1278.129123	217	2123
gene mutat	gene mutations	gene mutations|&|gene mutation|&|mutated genes|&|mutations in the gene|&|mutations in genes|&|mutations in the genes|&|mutation in the gene|&|mutations in these genes|&|mutations in a gene|&|mutations in this gene|&|mutation of the gene|&|	2	925.2999533	1.164356436	3148.663164	505	3646
gene product	gene products	gene products|&|gene product|&|product of the gene|&|product of this gene|&|product of a gene	2	420.1617135	1.076612903	1469.73399	248	1181
gene regul	gene regulation	gene regulation|&|regulation of gene|&|regulation gene|&|regulation of the gene|&|gene in the regulation	2	626.3084718	1.081521739	6567.413977	368	1733
gene target	target genes	target genes|&|target gene|&|gene target|&|genes target	2	1122.004875	1.170771757	8637.791229	609	3398
gener popul	general population	general population	2	1450.895505	1.340116279	1376.256574	688	7376
gener practic	general practices	general practices|&|general practice	2	492.5491248	1.257028112	1299.707951	249	2770
genet variant	genetic variants	genetic variants	2	473.6654523	1.148854962	2889.817836	262	2059
genet variat	genetic variation	genetic variation|&|Genetic variation	2	755.3469007	1.153846154	1542.025744	416	2361
genom human	human genome	human genome	2	786.8196882	1.157407407	1854.239533	432	1851
ground state	ground state	ground state	2	412.2935166	1.207373272	2183.846029	217	639
group intervent	intervention group	intervention group|&|intervention groups|&|group intervention|&|groups after intervention	2	1696.383248	2.191056911	8518.82803	492	5691
group oncolog	Oncology Group	Oncology Group|&|oncology group|&|group in oncology	2	695.5486044	1.023148148	6369.303915	1159	15787
group oncolog perform statu	Oncology Group performance status	Oncology Group performance status	4	575.8564243	1.003460208	10391.98993	289	6183
group placebo	placebo group	placebo group|&|placebo groups|&|groups versus placebo	2	5181.994466	2.015299878	6307.434894	1664	22594
group studi	study groups	study groups|&|study group|&|Study Group|&|Group Study|&|group study|&|groups in this study|&|groups during the study|&|group of the study	2	1241.601468	1.082304527	1327.619507	903	9038
group treatment	treatment groups	treatment groups|&|treatment group|&|Treatment Group|&|treatment in each group|&|groups of treatment|&|treatment in this group|&|treatment for this group|&|groups after treatment|&|treatment in all groups|&|treatment in both groups|&|treatment	2	2354.164507	1.290767903	3173.922569	1227	16431

group vs	group vs	group vs|&|vs group|&|group I vs	2	1055.912022	1.266037736	3857.664302	530	8931
growth rate	growth rate	growth rate|&|growth rates|&|rate of growth|&|rates of growth	2	486.2545673	1.282157676	1568.940581	241	968
growth tumor	tumor growth	tumor growth|&|growth of a tumor|&|growth of tumor	2	621.5875537	1.196969697	3196.987456	330	2587
h3 histon	histone H3	histone H3|&|H3 histone	2	640.4712262	1.222222222	6634.118257	454	2380
hazard ratio	hazard ratio	hazard ratio|&|hazard ratios|&|Hazard ratios	2	8576.334601	1.195699868	6777.159532	5127	75015
head neck	head and neck	head and neck|&|head or neck	3	440.6190254	1.233480176	3892.454476	227	2814
health servic	health services	health services|&|Health Service|&|health service|&|Service Health	2	703.4168012	1.214673913	1570.399524	439	3598
health studi	Health Study	Health Study|&|Study of Health|&|Study Health	2	402.8516804	1.066666667	3027.092266	240	3127
health system	health systems	health systems|&|health system	2	695.5486044	1.351681957	1958.962438	327	2232
heart rate	heart rate	heart rate	2	996.1137252	1.555282555	2431.831496	458	4454
helper t	T helper	T helper|&|helper T	2	391.8362047	1.055084746	8468.723757	236	1409
hemispher northern	Northern Hemisphere	Northern Hemisphere	2	410.7198772	1.266990291	7384.345935	206	861
hg mm	mm Hg	mm Hg	2	1136.16763	1.675174014	7370.479083	431	5799
higher risk	higher risk	higher risk	2	1186.52409	1.15467075	1625.583836	653	8632
histocompat major	major histocompatibility	major histocompatibility	2	421.7353529	1.015151515	4965.55189	264	1554
histon modif	histone modifications	histone modifications|&|histone modification	2	420.1617135	1.247663551	4171.324634	214	1267
hiv infect	HIV infection	HIV infection|&|infection with HIV	2	963.0672983	1.507389163	4257.05299	406	3322
hiv-1 infect	HIV-1 infection	HIV-1 infection|&|infection with HIV-1|&|infection by HIV-1	2	613.7193568	1.418181818	2351.192592	275	1899
human immunodefici viru	human immunodeficiency virus	human immunodeficiency virus	3	627.4849313	1.003174603	31706.46525	315	3273
ice sheet	ice sheet	ice sheet|&|ice sheets|&|Ice Sheet	2	723.8741131	1.660649819	3710.504034	316	1283
ii phase	phase II	phase II|&|Phase II	2	1798.669807	1.089609152	23841.18902	2073	23390
ii phase studi	phase II study	phase II study|&|Phase II Study|&|Phase II study|&|phase II studies|&|study phase II|&|Study Phase II	3	1693.812172	1.05308642	18618.82464	810	9778
ii pol	Pol II	Pol II	2	423.3089922	2.223140496	20056.95909	121	754
ii polymeras rna	RNA polymerase II	RNA polymerase II	3	687.0562855	1.098412698	89507.57825	315	1854
ii stage	stage II	stage II|&|II stage	2	709.7113587	1.523648649	1207.505027	296	3917
iii phase	phase III	phase III|&|Phase III	2	1875.778137	1.065236819	5229.383246	1704	19390
iii phase random	randomized phase III	randomized phase III|&|Randomized Phase III|&|phase III randomized|&|Phase III Randomized	3	416.9994797	1.019417476	5528.068322	206	2437
iii phase studi	phase III study	phase III study|&|Phase III Study	3	647.3420494	1.03164557	8170.424609	316	4073
iii stage	stage III	stage III	2	859.2070995	1.309352518	1901.542131	522	7086
ill patient	ill patients	ill patients	2	434.3244679	1.373134328	2486.578587	201	2031
immun innat	innate immunity	innate immunity	2	413.867156	1.095833333	1218.64311	241	1414
immun respons	immune responses	immune responses|&|immune response	2	1528.003834	1.165666267	8234.773081	906	5783
immun system	immune system	immune system	2	774.2305732	1.138888889	4600.933259	432	2516
import role	important role	important role|&|important roles	2	1400.539045	1.009070295	1722.200683	882	4813
incid rate	incidence rate	incidence rate|&|incidence rates|&|rates of incidence|&|incidence and rates	2	986.671889	1.38410596	5637.64826	579	7529
increas risk	increased risk	increased risk|&|increase the risk|&|increased risks|&|increase risk|&|increases the risk|&|increase in risk|&|increases risk|&|risk increase|&|increase in the risk|&|risk increases|&|increases in risk|&|increases in the risk|&|increases this risk|&|increas	2	5213.467254	1.340210356	5630.079825	2654	31996
independ predictor	independent predictor	independent predictor|&|independent predictors	2	1087.384809	1.153589316	3350.780141	599	7657
induct therapi	induction therapy	induction therapy|&|therapy induction	2	428.0299104	1.353233831	2012.257531	201	2586
infarct myocardi	myocardial infarction	myocardial infarction|&|Myocardial Infarction	2	4832.646525	1.34989011	8721.981774	2749	30881
infarct myocardi non-fat	non-fatal myocardial infarction	non-fatal myocardial infarction	3	415.0137679	1.244047619	1822.105107	168	2344
infect viru	virus infection	virus infection|&|infection by this virus|&|infection with this virus	2	621.5875537	1.278317152	1131.911503	309	2372
inform quantum	quantum information	quantum information|&|quantum of information	2	657.7812593	1.197707736	3366.529279	349	853
inhibitor kinas	kinase inhibitor	kinase inhibitor|&|kinase inhibitors|&|inhibitor of the kinase	2	465.7972554	1.203252033	12568.75484	539	6959
inhibitor kinas tyrosin	tyrosine kinase inhibitor	tyrosine kinase inhibitor|&|tyrosine kinase inhibitors	3	643.3706258	1.08361204	30042.76167	299	4739
in-hospit mortal	in-hospital mortality	in-hospital mortality|&|In-hospital mortality	2	613.7193568	1.652542373	1859.854477	236	2986
insight new	new insights	new insights|&|new insight	2	572.804733	1.005524862	2233.370216	362	1953
insulin resist	insulin resistance	insulin resistance	2	1018.144677	1.58190709	1190.753744	409	3054
intention-to-treat popul	intention-to-treat population	intention-to-treat population	2	700.2695225	1.138107417	5574.789654	486	8000
interact protein-protein	protein-protein interactions	protein-protein interactions|&|protein-protein interaction	2	484.6809279	1.12	2109.932436	275	1281
interfer rna	RNA interference	RNA interference|&|interference RNA	2	939.4627077	1.043706294	7780.567764	572	2785
interquartil rang	interquartile range	interquartile range	2	915.8581171	1.063985375	3171.826694	547	7744
intervent patient	Interventions Patients	Interventions Patients|&|INTERVENTIONS Patients|&|Intervention Patients	2	517.7273548	1	5543.404679	329	5206
iv stage	stage IV	stage IV	2	508.2855186	1.281746032	1640.918304	300	4326
key role	key role	key role|&|key roles	2	859.2070995	1.009242144	1894.24059	541	2566
kinas protein	protein kinase	protein kinase|&|protein kinases|&|kinase protein	2	1403.686324	1.105328377	5992.572687	1017	5890
kinas receptor tyrosin	receptor tyrosine kinase	receptor tyrosine kinase|&|receptor tyrosine kinases|&|tyrosine kinase receptor	3	589.7564069	1.060714286	22737.52415	280	2563
kinas tyrosin	tyrosine kinase	tyrosine kinase|&|tyrosine kinases	2	613.7193568	1.147058824	13635.54639	828	8771
kingdom unit	United Kingdom	United Kingdom	2	690.8276862	1.131443299	1416.919977	388	4215
lack mice	mice lacking	mice lacking|&|Mice lacking	2	434.3244679	1.045454545	1479.98749	264	1323
length scale	length scales	length scales|&|length scale|&|scale length	2	404.4253197	1.13215859	4295.315005	227	526
level protein	protein levels	protein levels|&|protein level|&|level of the protein|&|levels of protein|&|level of protein	2	479.9600098	1.159695817	1147.846367	313	2666
level sea	sea level	sea level	2	393.4098441	1.453488372	3249.153737	172	588
life qualiti	quality of life	quality of life|&|Quality of life|&|life quality|&|life and quality	3	2492.644772	1.290953545	1647.271772	1227	13506
ligas ubiquitin	ubiquitin ligase	ubiquitin ligase|&|ubiquitin ligases	2	522.448273	1.133105802	3272.028136	439	2319
logist regress	logistic regression	logistic regression|&|Logistic regression	2	582.2465693	1.010928962	1548.497088	433	5013
loss weight	weight loss	weight loss|&|loss of both weight|&|loss of weight	2	1148.756745	1.784841076	2320.92121	409	4313
lower rate	lower rates	lower rates|&|lower rate	2	689.2540469	1.140625	1299.515648	384	4852
lower risk	lower risk	lower risk	2	959.9200196	1.252566735	1971.421675	487	6746
lymph node	lymph nodes	lymph nodes|&|lymph node	2	1686.941411	1.436997319	2966.189	1019	11175
lymphocyt t	T lymphocytes	T lymphocytes|&|T lymphocyte	2	572.804733	1.166666667	6587.151454	370	2355
magnitud order	orders of magnitude	orders of magnitude|&|order of magnitude|&|orders magnitude|&|orders in magnitude|&|order and magnitude	3	1013.423758	1.022222222	2669.11333	630	1604
main measur outcom	Main Outcome Measures	Main Outcome Measures|&|Main outcome measures|&|Main Outcome Measure|&|MAIN OUTCOMES AND MEASURES|&|Main outcome measure|&|MAIN OUTCOME MEASURES|&|MAIN OUTCOME AND MEASURES|&|MAIN OUTCOMES ME	3	9451.988206	1	57553.74052	4760	59133
main outcom	Main Outcome	Main Outcome|&|Main outcome|&|MAIN OUTCOMES	2	487.8282067	1	57480.94422	4928	61220
mass spectrometri	mass spectrometry	mass spectrometry	2	922.1526746	1.087198516	2012.493497	596	2619
massachusett medic societi	Massachusetts Medical Society	Massachusetts Medical Society	3	1348.298318	1.001474926	52783.45096	678	6752
measur outcom	Outcome Measures	Outcome Measures|&|outcome measures|&|Outcome Measure|&|outcome measure|&|OUTCOME MEASURES|&|OUTCOME MEASURE|&|measures of outcome|&|measure of outcome	2	1077.942973	1.078740157	48696.18183	4913	57888
measur outcom primari	primary outcome measure	primary outcome measure|&|MEASURES The primary outcome|&|outcome measures Primary|&|Primary outcome measures|&|measures Primary outcome	3	1028.598716	1.017681729	7783.428814	509	6695
mechan molecular	molecular mechanisms	molecular mechanisms|&|molecular mechanism	2	1441.453669	1.024608501	5567.256605	894	4362
mechan underli	underlying mechanisms	underlying mechanisms|&|mechanisms underlying|&|underlying mechanism|&|mechanism underlying	2	901.6953627	1.021390374	2549.223198	561	3030
mechan ventil	mechanical ventilation	mechanical ventilation	2	539.7583061	1.625592417	4716.435256	211	2583
median os	median OS	median OS|&|Median OS	2	457.9290585	1.127906977	3690.162113	258	4385
median pf	median PFS	median PFS|&|Median PFS	2	572.804733	1.170418006	5639.39104	311	5616

median progression-fre	median progression-free	median progression-free|&|Median progression-free	2	873.3698539	1.065259117	6152.888246	521	8967
median time	median time	median time|&|Median time	2	1167.640417	1.086383602	3154.216903	697	9753
membran plasma	plasma membrane	plasma membrane	2	1065.353858	1.306949807	5065.146493	518	1988
membran protein	membrane proteins	membrane proteins|&|membrane protein|&|proteins in the membrane|&|protein within the membrane|&|protein and membrane	2	1066.927497	1.25323475	4619.179522	541	2289
men women	men and women	men and women|&|women and men|&|women than men|&|men than women|&|men or women|&|men versus women|&|women vs. men|&|women or men|&|men women|&|men nor women|&|women as men|&|men vs. women	3	1013.423758	1.311608961	2565.496164	491	5658
messeng rna	messenger RNA	messenger RNA|&|messenger RNAs	2	1274.647895	1.024020228	7808.235292	791	3868
meta-analysi review systemat	systematic review and meta-analysis	systematic review and meta-analysis|&|Systematic review and meta-analysis|&|systematic review with meta-analysis	4	742.6562162	1.021857924	24992.31766	366	3895
metastat patient	patients with metastatic	patients with metastatic|&|metastatic patients|&|patients metastatic	3	428.0299104	1.142857143	2665.490624	239	3298
method patient	Patients and Methods	Patients and Methods|&|Methods Patients|&|Methods All patients|&|patients and Methods|&|Methods patients|&|Methods The patients|&|patients Methods|&|Methods Of the patients|&|Methods Although patients|&|Methods From patients|&|Method	3	10419.06631	1	27125.52224	6721	85087
method result	Methods and results	Methods and results|&|Methods and Results|&|Methods Results	3	3417.944725	1	15447.51737	2172	21858
mice transgen	transgenic mice	transgenic mice	2	574.3783724	1.137071651	1292.846374	321	1529
mice wild-typ	wild-type mice	wild-type mice|&|mice from wild-type|&|mice with a wild-type	2	398.1307622	1.090517241	1419.058588	232	1176
missens mutat	missense mutations	missense mutations|&|missense mutation	2	597.982963	1.111111111	1695.878603	342	1693
model mous	mouse model	mouse model|&|mouse models|&|mouse as a model|&|mouse that models	2	2251.877948	1.096551724	4100.273889	1305	7260
model regress	regression models	regression models|&|regression model|&|model regression	2	827.734312	1.021359223	2492.702715	771	9919
model system	model system	model system|&|model systems|&|systems model|&|system model|&|model of the system	2	597.982963	1.016042781	1299.35798	374	1347
molecul small	small molecules	small molecules|&|small molecule	2	550.7737817	1.118210863	1549.549588	313	1414
mortal rate	mortality rates	mortality rates|&|mortality rate|&|Mortality rates|&|rates of mortality|&|rate of mortality|&|mortality during rate|&|Mortality and rates|&|mortality or rates	2	2413.962803	1.568507157	2270.607766	978	11476
mortal risk	mortality risk	mortality risk|&|risk of mortality|&|risk for mortality|&|mortality and risk|&|risk mortality	2	489.4018461	1.264227642	1427.997604	271	3574
multipl myeloma	multiple myeloma	multiple myeloma|&|Multiple Myeloma	2	730.1686706	1.230769231	1760.958088	418	3824
multipl sclerosi	multiple sclerosis	multiple sclerosis	2	1206.981402	1.779582367	1196.114575	431	2915
mutat point	point mutations	point mutations|&|point mutation	2	585.393848	1.110447761	1130.641065	335	1875
myelodysplast syndrom	myelodysplastic syndrome	myelodysplastic syndrome|&|myelodysplastic syndromes	2	442.1926648	1.151639344	5343.210279	244	2562
natriuret peptid	natriuretic peptide	natriuretic peptide|&|natriuretic peptides	2	404.4253197	1.241545894	2192.353238	394	4091
nervous system	nervous system	nervous system	2	456.3554192	1.062271062	1973.556982	274	1075
object rate respons	objective response rate	objective response rate|&|rate of objective response	3	756.5561988	1.168711656	14338.78687	326	6199
object respons	objective response	objective response|&|objective responses|&|Objective response	2	945.7572652	1.362811791	9819.618915	712	12410
observ report	report observations	report observations|&|report the observation|&|report on the observation|&|report observation|&|report on observations|&|report an observation|&|report on observation	2	527.1691911	1	6880.290621	335	757
observ studi	observational study	observational study|&|observational studies|&|Observational study	2	1079.516612	1.106451613	1309.829848	621	6325
ocean southern	Southern Ocean	Southern Ocean	2	393.4098441	1.666666667	8094.046244	150	666
odd ratio	odds ratio	odds ratio|&|odds ratios|&|Odds ratios|&|ratio of odds	2	5328.342928	1.1740638	7016.746892	3331	36911
onset symptom	symptom onset	symptom onset|&|onset of symptoms|&|symptoms onset|&|onset of the symptoms|&|onset symptoms	2	514.5800761	1.318548387	1112.074861	248	2929
option treatment	treatment options	treatment options|&|treatment option|&|options for treatment|&|option for treatment|&|option for the treatment	2	923.7263139	1.055755396	1490.601566	556	7228
outcom primari	primary outcome	primary outcome|&|primary outcomes|&|Primary outcomes|&|Primary outcome|&|outcomes Primary	2	4207.911692	1.184758529	12277.54741	2421	29018
outcom secondari	Secondary outcomes	Secondary outcomes|&|secondary outcomes|&|secondary outcome	2	1819.127119	1.119070668	12331.48955	1033	14021
overal rate respons	overall response rate	overall response rate|&|overall rate of response|&|rate of overall response	3	935.2702615	1.087759815	10135.03243	433	6827
overal rate surviv	overall survival rates	overall survival rates|&|overall survival rate	3	510.3279346	1.122270742	1962.330109	229	3089
oxid stress	oxidative stress	oxidative stress	2	635.7503081	1.320261438	1106.983539	306	2075
oxygen reactiv speci	reactive oxygen species	reactive oxygen species|&|reactive species of oxygen	3	613.5849486	1.084210526	1540.371741	285	1550
p trend	P for trend	P for trend|&|P trend|&|trend P	3	413.867156	1.43715847	1654.432917	183	2572
partial respons	partial response	partial response|&|partial responses	2	1652.321345	1.101783841	18473.48655	953	14936
pathway signal	signaling pathway	signaling pathway|&|signaling pathways|&|signalling pathway|&|signalling pathways|&|pathway signaling	2	1669.631378	1.079348932	6872.839022	983	5861
patient proport	proportion of patients	proportion of patients|&|proportions of patients|&|proportion of all patients|&|proportion of the patients|&|proportions of all patients|&|proportion of these patients	3	1312.41524	1.176304654	2368.810184	709	9507
patient receiv	patients receiving	patients receiving|&|Patients receiving	2	1894.661809	1.188548865	1187.335102	1074	14460
patient undergo	patients undergoing	patients undergoing	2	923.7263139	1.181086519	2374.909342	559	6720
percentag point	percentage points	percentage points|&|points in the percentage	2	761.6414582	1.49382716	1324.782201	324	3793
perform statu	performance status	performance status	2	1057.485661	1.195729537	6509.199207	900	15753
period studi	study period	study period|&|period of study|&|period under study|&|study during the period|&|period of the study	2	876.5171326	1.127530364	1174.807315	494	5535
phase s	S phase	S phase	2	428.0299104	1.353233831	13340.49009	201	1103
phase transit	phase transition	phase transition|&|phase transitions	2	741.1841463	1.326760563	3395.826512	355	1063
placebo vs	vs placebo	vs placebo|&|placebo vs	2	453.2081404	1.447236181	2529.977545	199	3015
polymeras rna	RNA polymerase	RNA polymerase	2	720.7268344	1.153652393	8143.472602	705	3588
polymorph single-nucleotid	single-nucleotide polymorphisms	single-nucleotide polymorphisms|&|single-nucleotide polymorphism	2	516.1537155	1.041269841	2964.409061	315	2223
postmenopaus women	postmenopausal women	postmenopausal women	2	920.5790352	1.392857143	1855.994547	420	5293
primari tumor	primary tumor	primary tumor|&|primary tumors	2	637.3239474	1.306451613	1791.716892	310	3395
profil safeti	safety profile	safety profile	2	608.9984387	1.057377049	3429.762106	430	6804
prognost signific	prognostic significance	prognostic significance	2	539.7583061	1.124590164	2210.483456	305	3771
prognost valu	prognostic value	prognostic value|&|Prognostic value	2	1133.020351	1.208053691	1782.215847	596	7258
progress tumor	tumor progression	tumor progression|&|progression of tumor	2	453.2081404	1.205020921	1169.610409	239	2471
propens score	propensity score	propensity score	2	393.4098441	1.256281407	1270.37547	199	2809
prostatectomi radic	radical prostatectomy	radical prostatectomy	2	525.5955517	1.582938389	4353.209987	211	2525
protein synthesi	protein synthesis	protein synthesis|&|synthesis of protein	2	632.6030293	1.260188088	4821.023162	319	1443
purpos studi	purpose of this study	purpose of this study|&|Purpose This study|&|purpose of the study|&|Purpose In this study|&|study purpose|&|Purpose The study	4	615.2929962	1	1102.470148	391	4672
quantum state	quantum states	quantum states|&|quantum state|&|state quantum|&|state as a quantum	2	497.2700429	1.253968254	1288.15766	252	651
radiat therapi	radiation therapy	radiation therapy|&|Radiation Therapy	2	1595.670328	1.293367347	3060.764295	883	9778
rang wide	wide range	wide range	2	1260.48514	1.024296675	1474.639499	782	3346
rate ratio	rate ratio	rate ratio|&|rate ratios|&|ratio of the rate	2	889.1062477	1.358173077	5447.110719	544	6513
rate respons	response rate	response rate|&|response rates|&|Response rates|&|rate of response|&|rates of response|&|rate response|&|response than the rate	2	2944.279273	1.294809689	16665.39599	2044	29756
rate surviv	survival rates	survival rates|&|survival rate	2	1391.097209	1.210958904	2480.849374	791	10244
receptor toll-lik	Toll-like receptor	Toll-like receptor|&|Toll-like receptors|&|receptor Toll-like|&|receptors Toll-like	2	462.6499767	1.035211268	5299.882638	284	1676
regul transcript	transcriptional regulation	transcriptional regulation|&|transcriptional regulators	2	428.0299104	1.038167939	2851.634753	262	1406
rel risk	relative risk	relative risk|&|relative risks|&|Relative risks	2	3575.308663	1.319396051	4634.639076	1770	21242
research treatment	Research and Treatment	Research and Treatment	3	446.9135829	1.010676157	3939.247115	281	3595
respons tumor	tumor response	tumor response|&|response by tumor|&|response of the tumor	2	481.5336492	1.26446281	2406.751	247	3586
result show	results show	results show|&|show results|&|show that this results|&|show that the results|&|show the results|&|show that these results|&|show that it results|&|results We show|&|results and show	2	1343.888027	1.004705882	1558.160229	850	3813
review systemat	systematic review	systematic review|&|Systematic review|&|systematic reviews	2	885.9589689	1.172916667	5356.182203	908	8629
rhythm sinu	sinus rhythm	sinus rhythm	2	421.7353529	1.604790419	3685.570589	167	1758
risk stratif	risk stratification	risk stratification|&|stratification of risk	2	750.6259825	1.15496368	1200.37519	413	4442
risk stroke	stroke risk	stroke risk|&|risk of stroke|&|risk for stroke|&|risk of a stroke	2	445.3399435	1.429292929	1470.493762	198	2312
room temperatur	room temperature	room temperature	2	719.153195	1.120098039	4924.277658	408	1056
scale time	time scales	time scales|&|time scale	2	467.3708948	1.072202166	1971.858316	277	679
sea surfac temperatur	sea surface temperature	sea surface temperature|&|sea surface temperatures	3	444.799445	1.317647059	5341.51801	170	782

signal transduct	signal transduction	signal transduction	2	418.5880741	1.090163934	2299.119035	244	1200
size tumor	tumor size	tumor size|&|size of tumor|&|size of the tumor	2	580.6729299	1.294736842	2486.420613	285	3984
solar system	Solar System	Solar System|&|solar system	2	599.5566024	1.27	1312.008577	300	502
solid tumor	solid tumors	solid tumors|&|Solid Tumors	2	739.6105069	1.143552311	2922.753113	565	7375
state unit	United States	United States	2	3968.718507	1.253479125	5645.002701	2012	19450
stem-cel transplant	stem-cell transplantation	stem-cell transplantation|&|Stem-Cell Transplantation	2	508.2855186	1.12543554	2429.971881	678	7213
stent thrombosi	stent thrombosis	stent thrombosis	2	610.572078	1.694323144	4675.138996	229	2904
suppressor tumor	tumor suppressor	tumor suppressor|&|tumor suppressors	2	756.92054	1.190594059	4352.263287	535	3377
suppressor tumour	tumour suppressor	tumour suppressor	2	440.6190254	1.212121212	2873.604028	231	1524
